# Prevalence of common disease-associated variants in Asian Indians

**DOI:** 10.1186/1471-2156-9-13

**Published:** 2008-02-04

**Authors:** Trevor J Pemberton, Niyati U Mehta, David Witonsky, Anna Di Rienzo, Hooman Allayee, David V Conti, Pragna I Patel

**Affiliations:** 1 Institute for Genetic Medicine, Keck School of Medicine, University of Southern California, Los Angeles, CA, USA; 2 Department: of Preventive Medicine, Keck School of Medicine, University of Southern California, Los Angeles, CA, USA; 3 Department of Biochemistry and Molecular Biology, University of Southern California, Los Angeles, CA, USA; 4 Center for Craniofacial Molecular Biology, Keck School of Medicine, University of Southern California, Los Angeles, CA, USA; 5 Department of Human Genetics, University of Chicago, Chicago, IL, USA

## Abstract

**Background:**

Asian Indians display a high prevalence of diseases linked to changes in diet and environment that have arisen as their lifestyle has become more westernized. Using 1200 genome-wide polymorphisms in 432 individuals from 15 Indian language groups, we have recently shown that: (i) Indians constitute a distinct population-genetic cluster, and (ii) despite the geographic and linguistic diversity of the groups they exhibit a relatively low level of genetic heterogeneity.

**Results:**

We investigated the prevalence of common polymorphisms that have been associated with diseases, such as atherosclerosis (*ALOX5*), hypertension (*CYP3A5*, *AGT*, *GNB3*), diabetes (*CAPN10*, *TCF7L2*, *PTPN22*), prostate cancer (DG8S737, rs1447295), Hirschsprung disease (*RET*), and age-related macular degeneration (*CFH*, *LOC387715*). In addition, we examined polymorphisms associated with skin pigmentation (*SLC24A5*) and with the ability to taste phenylthiocarbamide (*TAS2R38*). All polymorphisms were studied in a cohort of 576 India-born Asian Indians sampled in the United States. This sample consisted of individuals whose mother tongue is one of 14 of the 22 "official" languages recognized in India as well as individuals whose mother tongue is Parsi, a cultural group that has resided in India for over 1000 years. Analysis of the data revealed that allele frequency differences between the different Indian language groups were small, and interestingly the variant alleles of *ALOX5 *g.8322G>A and g.50778G>A, and *PTPN22 *g.36677C>T were present only in a subset of the Indian language groups. Furthermore, a latitudinal cline was identified both for the allele frequencies of the SNPs associated with hypertension (*CYP3A5*, *AGT*, *GNB3*), as well as for those associated with the ability to taste phenylthiocarbamide (*TAS2R38*).

**Conclusion:**

Although caution is warranted due to the fact that this US-sampled Indian cohort may not represent a random sample from India, our results will hopefully assist in the design of future studies that investigate the genetic causes of these diseases in India. Our results also support the inclusion of the Indian population in disease-related genetic studies, as it exhibits unique genotype as well as phenotype characteristics that may yield new insights into the underlying causes of common diseases that are not available in other populations.

## Background

India is currently the second most populous country in the world after China with a population of 1,087 million that is predicted to expand to 1,628 million by the year 2050 [1]. The high prevalence of endogamy and relatively low admixture present in the population distinguishes Asian Indians (from the subcontinental region comprising India, Pakistan, Bangladesh and Sri Lanka) from most other populations presently used in genetic studies [2]. As the country is becoming more westernized both in diet and lifestyle, the prevalence of diseases associated with these lifestyle changes is increasing.

The languages of India belong to four major families: Indo-Aryan (a branch of the Indo-European family), Dravidian, Austroasiatic, and Sino-Tibetan, with the overwhelming majority of the population speaking languages belonging to the first two families [3]. The 1981 census – the last census to tabulate languages – reported 112 mother tongues with more than 10,000 speakers and almost 1 million people speaking other languages. The Indian constitution recognizes 22 official Indian languages. They are Assamese, Bengali, Bodo, Dogri, Gujarati, Hindi, Kannada, Kashmiri, Konkani, Maithili, Malayalam, Manipuri, Marathi, Nepali, Oriya, Punjabi, Sanskrit, Santhali, Sindhi, Tamil, Telugu, and Urdu. Of the official languages, about 43 percent of the population speaks Hindi as their mother tongue. Telugu, Bengali, Marathi, and Tamil rank next, each the mother tongue of about 4 to 5 percent; Urdu, Gujarati, Malayalam, Kannada, and Oriya are claimed by between 2 and 3 percent; Bhojpuri, Punjabi, and Assamese by 1 to 2 percent; and all other languages by less than 1 percent each. Pertinent to this study, with the exception of Hindi, which is the primary language spoken in several north Indian states, each of the other languages is spoken by individuals with origins from a distinct Indian state [3] and hence, each corresponds to a geographically defined region within India.

The Parsis are a member of the close-knit Zoroastrian community found primarily in the Indian state of Gujarat. Although the Parsis of India are descendents from Persian Zoroastrians who emigrated to the Indian subcontinent from Persia over 1,000 years ago [4-6], they no longer have social or familial ties to Persians, and do not share language or recent history with them. Mitochondrial and Y-chromosome analyses suggest that since their arrival in India, the Parsis have integrated themselves into Indian society through a male-mediated migration of their ancestors who admixed with local females [7,8]. However, they have simultaneously maintained their own ethnic identity, making the Parsi community both Indian in terms of national affiliation, language and history, but not typically Indian in terms of consanguinity or cultural, behavioral and religious practices.

Large inter-ethnic variation is known for a number of common conditions, which may be due, in part, to differences in risk allele frequencies that are the result of either population structure, migration, or the action of natural selection on the risk variants themselves [9]. Because selective pressures may vary with geography and other environmental variables, detectable patterns of geographic variation may sometimes be observed in disease alleles. Recent population structure analysis using 1200 genome-wide polymorphisms in 432 individuals from 15 Indian language groups has shown that Indians constitute a distinct population cluster and that despite the geographic and linguistic diversity of the groups, they exhibit a low level of genetic differentiation [10].

Metabolic disorders have been found to have a disproportionately high prevalence in the Indian population, a phenomenon which is likely associated with the increasing westernization of India. For example, many studies have shown that the prevalence of coronary artery disease (CAD) in individuals of Indian origin is currently much higher than in other ethnic groups and is even increasing [11-17]. It is also currently estimated that 18% of the Indian population suffer from hypertension, one of the major risk factors associated with CAD, and that the prevalence of hypertension is also increasing within this population [18]. However, this prevalence is comparable to that in other worldwide populations (15.0–40.0%) [19]. Type-2 diabetes is now reaching epidemic proportions in India, most prominently in urban Indians where there has been a very steep increase in prevalence over the last decade from 8.3% in 1992, 11.6% in 1997, to 15.5% in 2005 [20-23]. Other diseases are also found at varying frequencies in the Indian population including Hirschsprung disease [24,25], age-related macular degeneration (AMD) [26,27], prostate cancer [28,29], and type-1 diabetes [30,31]. However, despite the high prevalence of common disorders and the large population within which to study them, most modern genetic studies have not incorporated Indian populations.

In the present study, we have investigated the prevalence of common polymorphisms that have recently been reported to be risk factors for atherosclerosis, hypertension, diabetes, prostate cancer, Hirschsprung disease, and age-related macular degeneration (Table 1) in a cohort of 576 India-born Asian Indians sampled in the United States. This cohort consists of individuals whose mother tongue is one of 14 of the 22 "official" languages recognized in India, as well as individuals whose mother tongue is Parsi. We have also investigated variation in the *TAS2R38 *bitter taste receptor, associated with the ability to taste phenylthiocarbamide (PTC), and in the *SLC24A5 *gene, associated with variation in human skin pigmentation (Table 1). The prevalence of these diseases/traits [32-43] and the risk-associated variant alleles of these polymorphisms (see Additional file 1) have been reported to vary between populations/ethnicities, suggesting that their influence may also vary between populations. The etiology of most cases of CAD [44], type-1 [45] and type-2 [46] diabetes, hypertension [47], prostate cancer [48], and AMD [49] are complex and are likely caused by the combined effect of genes and environment. However, the relative contribution of genes versus environment to the diseases/traits and the degree of genetic heterogeneity are likely very different for the different diseases/traits considered in this paper. Whilst we have investigated the prevalence of these minor alleles within this Indian cohort and compared the frequencies to those of other populations, we have not addressed the potential impact of environmental factors that may differ between these populations. This study will hopefully aid in the design of future genetic studies investigating the underlying causes of these diseases in India.

**Table 1:** Investigated polymorphisms and their associated diseases.

**Gene Name**	**Nucleotide Change**	**Protein Change**	**NCBI Acc. #**	**Phenotype**	**Reference**
*AGT*	g.802C>T	M235T	rs699	Hypertension	[100]
*CYP3A5*	g.6980G>A	*IVS4*	rs776746	Hypertension	[101]
*GNB3*	g.4423C>T	S275S	rs6489738	Hypertension	[53]
*ALOX5*	(5'-GGGCGG-3')_3–8_	*promoter*	*none*	Athersclerosis	[57]
*ALOX5*	g.20C>T	T7T	rs4987105	Athersclerosis	
*ALOX5*	g.8322G>A	T90T	rs2228064	Athersclerosis	
*ALOX5*	g.50778G>A	E243K	rs2228065	Athersclerosis	
*CAPN10*	g.4834T>C	*IVS3*	rs2975760	Type-2 diabetes	[102]
*TCF7L2*	g.98386G>T	*IVS4*	rs12255372	Type-2 diabetes	[103]
*PTPN22*	g.36677C>T	R620W	rs2476601	Type-1 diabetes	[104]
*Intragenic*	(5'-AC-3')_13–30_	*none*	DG8S737	Prostate Cancer	[58]
*Intragenic*	C>A	*none*	rs1447295	Prostate Cancer	[58]
*CFH*	g.37989T>C	Y402H	rs1061170	Age-related macular degeneration	[105-107]
*LOC387715*	g.205G>T	A69S	rs10490924	Age-related macular degeneration	[108]
*RET*	g.9349G>A	*IVS1*	rs2435357	Hirschsprung disease	[109]
*TAS2R38*	g.144G>C	A49P	rs713598	ability to taste PTC	[59]
*TAS2R38*	g.784C>T	A262V	rs1726866	ability to taste PTC	[59]
*TAS2R38*	g.885A>G	I296V	rs10246939	ability to taste PTC	[59]
*SLC24A5*	g.13233G>A	A111T	rs1426654	Skin pigmentation	[110]

## Results

**Figure 1:**
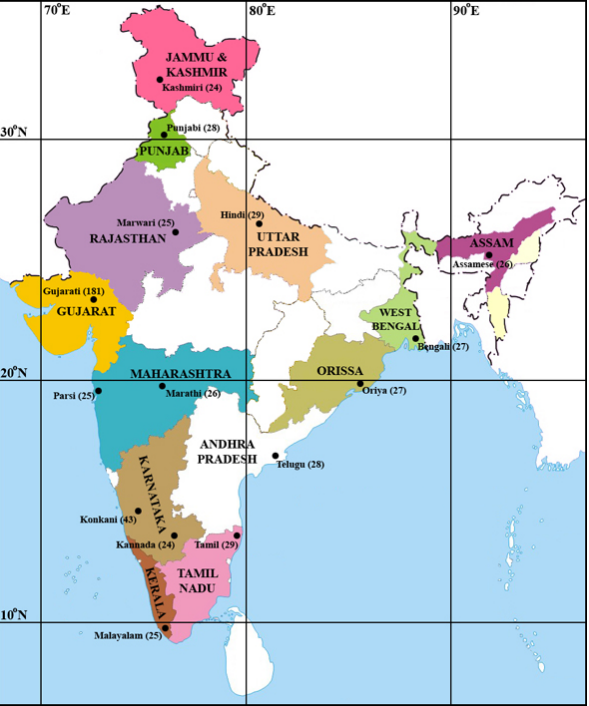
Sampled language groups, their sample sizes, and their geographic origins within India.

We have examined the variation of 19 common polymorphisms (Table 1) associated with diseases/traits in a collection of 576 individuals of Indian descent sampled in the United States that represent 14 of the 22 official or scheduled Indian languages as well as one additional cultural group (Parsi; Figure 1). These individuals are part of an expanded cohort that represents 15 groups defined by language and that was used in a previous population-genetic study [10]. The allele frequencies of these 19 common polymorphisms in the Indian cohort can be found in Tables 2, 3, 4; for genotype frequencies see Additional file 2, Tables S1–3.

**Table 2:** Allele frequencies of the polymorphisms associated with the metabolic disorders of diabetes, hypertension, and atherosclerosis.

			**Diabetes**			**Hypertension**			**Atherosclerosis**								
**Gene Name:**			**PTPN22**	**CAPN10**	**TCF7L2**	**CYP3A5**	**AGT**	**GNB3**	**ALOX5**						**ALOX5**	**ALOX5**	**ALOX5**
**Nucleotide Change:**			**g.36677C>T**	**g.4834T>C**	**g.98386G>T**	**g.6980G>A**	**g.802C>T**	**g.4423C>T**	**MS (5'-GGGCGG-3' starting at -145)**						**g.20C>T**	**g.8322G>A**	**g.50778G>A**
	***n***	**°N**	**T**	**C**	**T**	**A**	**T**	**T**	**3**	**4**	**5**	**6**	**7**	**8**	**T**	**A**	**A**

**Mother Tongue**																	
Assamese	26	26.00	0.000	0.220	0.280	0.220	0.400	0.280	0.000	0.160	0.800	0.040	0.000	0.000	0.140	0.000	0.020
Bengali	27	23.00	0.017	0.224	0.138	0.207	0.414	0.293	0.000	0.276	0.655	0.069	0.000	0.000	0.276	0.017	0.000
Gujarati	181	23.00	0.000	0.167	0.167	0.313	0.229	0.396	0.000	0.167	0.792	0.042	0.000	0.000	0.188	0.000	0.000
Hindi	29	30.30	0.012	0.209	0.244	0.221	0.395	0.279	0.000	0.186	0.767	0.047	0.000	0.000	0.186	0.012	0.000
Kannada	24	15.00	0.000	0.268	0.214	0.339	0.339	0.411	0.000	0.179	0.732	0.089	0.000	0.000	0.179	0.036	0.000
Kashmiri	24	32.44	0.000	0.320	0.480	0.080	0.480	0.420	0.000	0.180	0.800	0.020	0.000	0.000	0.160	0.020	0.000
Konkani	43	15.00	0.000	0.115	0.192	0.346	0.308	0.288	0.000	0.080	0.860	0.060	0.000	0.000	0.096	0.058	0.000
Malayalam	25	10.00	0.000	0.130	0.222	0.333	0.426	0.315	0.000	0.148	0.759	0.093	0.000	0.000	0.148	0.000	0.000
Marathi	26	20.00	0.000	0.278	0.315	0.481	0.333	0.259	0.000	0.111	0.778	0.111	0.000	0.000	0.111	0.000	0.000
Marwari	25	27.00	0.006	0.265	0.243	0.265	0.423	0.296	0.008	0.186	0.758	0.044	0.000	0.003	0.193	0.019	0.003
Oriya	27	20.00	0.019	0.192	0.231	0.250	0.308	0.346	0.000	0.231	0.692	0.077	0.000	0.000	0.231	0.000	0.000
Parsi	25	19.00	0.020	0.280	0.180	0.240	0.320	0.440	0.000	0.240	0.660	0.100	0.000	0.000	0.240	0.020	0.000
Punjabi	28	30.40	0.017	0.155	0.241	0.259	0.379	0.345	0.034	0.259	0.690	0.017	0.000	0.000	0.241	0.000	0.017
Tamil	29	11.00	0.018	0.161	0.321	0.143	0.429	0.393	0.018	0.232	0.714	0.036	0.000	0.000	0.232	0.054	0.018
Telugu	28	16.00	0.000	0.229	0.271	0.167	0.354	0.229	0.000	0.229	0.688	0.063	0.021	0.000	0.229	0.000	0.000
																	
F_ST_			0.016	0.008	0.002	0	0.011	0.010	0.006						0.013	0.009	0.013
p-value			1	1	1	1	1	1	1						1	1	1

**Gender**																	
Female	237		0.002	0.241	0.241	0.295	0.390	0.316	0.008	0.191	0.733	0.066	0.002	0	0.192	0.017	0.006
Male	339		0.013	0.224	0.254	0.236	0.385	0.320	0.004	0.192	0.753	0.049	0	0.001	0.193	0.016	0.003
																	
F_ST_			0	0.041	0.046	0	0.052	0.052	0						0.052	0.052	0.037
p-value			1	0.839	0.830	1	0.820	0.820	1						0.819	0.820	0.847

**Population**																	
Cohort	576		0.009	0.231	0.248	0.260	0.387	0.319	0.006	0.192	0.745	0.056	0.001	0.001	0.193	0.016	0.004
Standard Error			0.002	0.016	0.021	0.025	0.017	0.017	0.003	0.014	0.015	0.007	0.001	1.85 × 10^-4^	0.013	0.005	0.002
																	
HWE Constant			4.98 × 10^-5^	0.003	0.001	0.013	0.004	0.001	0.009						0.016	2.50 × 10^-4^	1.94 × 10^-5^
p-value			1	0.720	1	0.100	0.658	1	0.213						**0.014**	1	1
																	
Welch modified t-test:																	
Africa				**5.57 × 10**^-9^		**8.44 × 10**^-3^	**4.55 × 10**^-10^									0.151	0.172
Europe			**7.16 × 10**^-6^	**0.003**	0.790	**3.19 × 10**^-4^	**1.57 × 10**^-4^	**0.036**								0.724	0.067
Middle East						**1.94 × 10**^-9^	**0.007**										
Central/South Asia			**0.006**	**4.65 × 10**^-5^	**1.16 × 10**^-6^	**4.89 × 10**^-14^	0.432	**1.37 × 10**^-7^								**0.007**	
East Asia						**4.64 × 10**^-10^	**1.19 × 10**^-9^										
Americas				**0.029**		**1.18 × 10**^-5^	**1.56 × 10**^-5^	0.136									
Oceania						**1.17 × 10**^-8^	**0.002**										

P-values significant at <0.05 are shown in **bold**.

°N = degrees of latitude north of the equator.

**Table 3:** Allele frequencies of the polymorphisms associated with prostate cancer.

		**Prostate Cancer**																		
**Polymorphism:**		**DG8S737**																		**rs1447295**
**Nucleotide Change:**		**MS**																		**C>A**
	***n***	**-10**	**-9**	**-8**	**-7**	**-6**	**-5**	**-4**	**-3**	**-2**	**-1**	**0**	**+1**	**+2**	**+3**	**+4**	**+5**	**+6**	**+7**	**A**

**Mother Tongue**																				
Assamese	26	0.000	0.040	0.000	0.000	0.000	0.020	0.120	0.020	0.060	0.180	0.220	0.100	0.060	0.080	0.080	0.020	0.000	0.000	0.120
Bengali	27	0.017	0.000	0.000	0.000	0.000	0.000	0.034	0.121	0.155	0.190	0.103	0.155	0.052	0.121	0.052	0.000	0.000	0.000	0.121
Gujarati	181	0.000	0.042	0.000	0.000	0.042	0.000	0.021	0.042	0.229	0.188	0.188	0.042	0.042	0.125	0.042	0.000	0.000	0.000	0.125
Hindi	29	0.023	0.023	0.000	0.012	0.012	0.012	0.128	0.035	0.151	0.209	0.116	0.093	0.035	0.070	0.023	0.035	0.023	0.000	0.186
Kannada	24	0.000	0.071	0.000	0.000	0.018	0.000	0.089	0.036	0.161	0.268	0.125	0.036	0.000	0.143	0.054	0.000	0.000	0.000	0.125
Kashmiri	24	0.000	0.000	0.000	0.000	0.000	0.000	0.060	0.060	0.120	0.220	0.120	0.200	0.040	0.120	0.060	0.000	0.000	0.000	0.040
Konkani	43	0.000	0.019	0.000	0.000	0.019	0.019	0.077	0.115	0.135	0.173	0.192	0.058	0.135	0.019	0.019	0.019	0.000	0.000	0.192
Malayalam	25	0.000	0.000	0.000	0.000	0.000	0.000	0.093	0.037	0.167	0.204	0.111	0.185	0.019	0.148	0.000	0.019	0.000	0.019	0.074
Marathi	26	0.000	0.000	0.000	0.000	0.037	0.000	0.074	0.037	0.093	0.278	0.111	0.130	0.056	0.093	0.056	0.037	0.000	0.000	0.111
Marwari	25	0.000	0.008	0.003	0.000	0.008	0.022	0.110	0.028	0.116	0.293	0.122	0.077	0.058	0.105	0.041	0.006	0.003	0.000	0.141
Oriya	27	0.000	0.000	0.019	0.019	0.058	0.038	0.058	0.135	0.077	0.173	0.173	0.096	0.038	0.077	0.019	0.019	0.000	0.000	0.308
Parsi	25	0.000	0.000	0.020	0.000	0.040	0.000	0.020	0.020	0.380	0.220	0.100	0.140	0.020	0.020	0.020	0.000	0.000	0.000	0.040
Punjabi	28	0.000	0.000	0.000	0.000	0.017	0.000	0.052	0.069	0.121	0.345	0.121	0.086	0.086	0.069	0.034	0.000	0.000	0.000	0.034
Tamil	29	0.000	0.000	0.000	0.000	0.018	0.018	0.161	0.018	0.125	0.143	0.143	0.143	0.054	0.107	0.054	0.018	0.000	0.000	0.161
Telugu	28	0.000	0.021	0.000	0.000	0.000	0.000	0.042	0.104	0.188	0.375	0.083	0.021	0.042	0.042	0.021	0.042	0.000	0.021	0.104
																				
F_ST_		0.007																		0.020
p-value		1																		1

**Gender**																				
Female	237	0.002	0.021	0.004	0.002	0.019	0.011	0.078	0.044	0.146	0.251	0.129	0.099	0.057	0.078	0.049	0.011	0.000	0.000	0.120
Male	339	0.003	0.009	0.001	0.001	0.013	0.013	0.091	0.052	0.140	0.242	0.133	0.094	0.049	0.105	0.032	0.013	0.004	0.003	0.136
																				
F_ST_		0																		0
p-value		1																		1

**Population**																				
Cohort	576	0.003	0.014	0.003	0.002	0.016	0.012	0.085	0.050	0.142	0.245	0.130	0.098	0.052	0.094	0.039	0.012	0.003	0.002	0.129
Standard Error		0.002	0.006	0.002	0.001	0.005	0.003	0.011	0.010	0.020	0.017	0.010	0.014	0.008	0.010	0.005	0.004	0.002	0.002	0.018
																				
HWE Constant		0.003																		0.002
p-value		0.433																		0.580
																				
Welch modified t-test:																				
Africa																				
Europe																				0.091
Asia																				0.486
Americas																				

Alleles of DG8S737 were assigned based upon the change in the number of repeats away from that of the reference sequence in the NCBI database (23 AC dinucleotide repeats). Allele -1 is equivalent to the prostate cancer risk-associated -8 allele reported by Amundadottir *et al*. that had 22 repeats [58]. P-values significant at <0.05 are shown in **bold**.

**Table 4:** Allele frequencies of the polymorphisms associated with melanogenesis, Hirschsprung disease, AMD, and the ability to taste PTC.

		**Melanogenesis**	**Hirschsprung**	**AMD**		**Ability to Taste PTC**		
**Gene Name:**		**SLC24A5**	**RET**	**CFH**	**LOC387715**	**TAS2R38**	**TAS2R38**	**TAS2R38**
**Nucleotide Change:**		**g.13242G>A**	**g.9349G>A**	**g.37989T>C**	**g.205G>T**	**g.144G>C**	**g.784C>T**	**g.885A>G**
	***n***	**G**	**T**	**C**	**T**	**C**	**C**	**G**

**Mother Tongue**								
Assamese	26	0.260	0.240	0.360	0.200	0.340	0.360	0.360
Bengali	27	0.293	0.207	0.276	0.397	0.310	0.310	0.310
Gujarati	181	0.208	0.271	0.333	0.375	0.313	0.313	0.313
Hindi	29	0.105	0.209	0.267	0.244	0.349	0.360	0.360
Kannada	24	0.268	0.232	0.304	0.464	0.196	0.250	0.250
Kashmiri	24	0.020	0.100	0.260	0.340	0.480	0.500	0.500
Konkani	43	0.058	0.250	0.385	0.269	0.308	0.308	0.308
Malayalam	25	0.259	0.259	0.222	0.500	0.259	0.296	0.296
Marathi	26	0.296	0.259	0.278	0.204	0.407	0.407	0.426
Marwari	25	0.041	0.273	0.265	0.334	0.384	0.398	0.406
Oriya	27	0.154	0.346	0.212	0.250	0.346	0.365	0.365
Parsi	25	0.100	0.300	0.300	0.380	0.280	0.280	0.300
Punjabi	28	0.052	0.293	0.431	0.414	0.328	0.310	0.328
Tamil	29	0.036	0.179	0.250	0.393	0.339	0.321	0.375
Telugu	28	0.000	0.188	0.167	0.354	0.417	0.417	0.417
								
F_ST_		0	0.011	0.009	0	0.009	0.010	0.011
p-value		1	1	1	1	1	1	1

**Gender**								
Female	237	0.110	0.232	0.268	0.348	0.331	0.338	0.342
Male	339	0.117	0.261	0.289	0.330	0.363	0.376	0.386
								
F_ST_		0.049	0.020	0.037	0.042	0.020	0.007	0
p-value		0.825	0.887	0.847	0.837	0.887	0.933	1

**Population**								
Cohort	576	0.114	0.249	0.280	0.338	0.350	0.360	0.368
Standard Error		0.028	0.015	0.018	0.024	0.018	0.017	0.017
								
HWE Constant		0.004	0.003	0.009	0.006	0.002	0.002	0.002
p-value		0.304	0.654	0.350	0.513	0.926	0.855	0.857
								
Welch modified t-test:								
Africa		**4.21 × 10**^-14^				**3.86 × 10**^-4^	**3.24 × 10**^-8^	**0.005**
Europe		**2.80 × 10**^-4^		**4.20 × 10**^-3^	0.068	**2.43 × 10**^-6^	**5.78 × 10**^-5^	**1.37 × 10**^-4^
Asia		**7.18 × 10**^-14^		**1.55 × 10**^-8^	**0.018**	0.055	**7.97 × 10**^-6^	**7.60 × 10**^-6^
Americas		**0.024**				**0.006**	**1.92 × 10**^-5^	**1.64 × 10**^-5^
Oceania						0.090	**0.009**	**0.003**

P-values significant at <0.05 are shown in **bold**.

### Indian language groups

All of the polymorphisms genotyped in our Indian cohort were found to be in Hardy-Weinberg equilibrium (HWE; Tables 2, 3, &4), except for the *ALOX5 *g.20C>T polymorphism which gave an HWE constant of 0.016 (p-value 0.014). However, when corrected for multiple testing using a Bonferroni correction, this deviation becomes insignificant (corrected p-value 0.266).

Computations of F_ST _suggested that there were no significant minor allele frequency (MAF) differences between the language groups (Tables 2, 3, &4). This is in agreement with a recent population structure analysis of this Indian cohort that found that the different language groups exhibited a low level of genetic differentiation [10]. Therefore, when comparing the MAF of these polymorphisms in this Indian cohort with their frequency in other populations, we grouped the MAFs of these polymorphisms in the Indian language groups into a single comparative group. Of those polymorphisms with MAF data reported in other populations (see Additional file 1, Tables S4–20), most had significant differences in frequency with our Indian cohort (Tables 2, 3, &4).

Patterns in the variation of the MAF of several of the 19 polymorphisms were visible between some of the language groups (Tables 2, 3, &4). Of note was the Kashmiri group, which had significantly higher MAFs than the other groups for the *CAPN10 *g.4834T>C and *TCF7L2 *g.98386G>T SNPs associated with type-2 diabetes risk (Table 2). Furthermore, considering the hypertension-associated SNPs, whilst the Kashmiri group had a significantly lower MAF for the *CYP3A5 *g.6980G>A SNP compared to the other groups, this language group also had the highest MAF for the *AGT *g.802C>T SNP and the second highest MAF for the GNB3g.4423C>T SNP (Table 2). Interestingly, whilst the Kashmiri group also had the highest MAFs for all three *TAS2R38 *SNPs associated with the ability to taste PTC (Table 4), the Kannada group had the lowest MAF for all three *TAS2R38 *SNPs. It was also noteworthy that the Oriya group had a significantly higher MAF of the rs1447295 SNP associated with prostate cancer risk than the other groups (Table 3), but this higher prevalence was not reflected in the DG8S737 microsatellite allele frequencies that are also associated with prostate cancer risk.

### Hypertension

**Figure 2:**
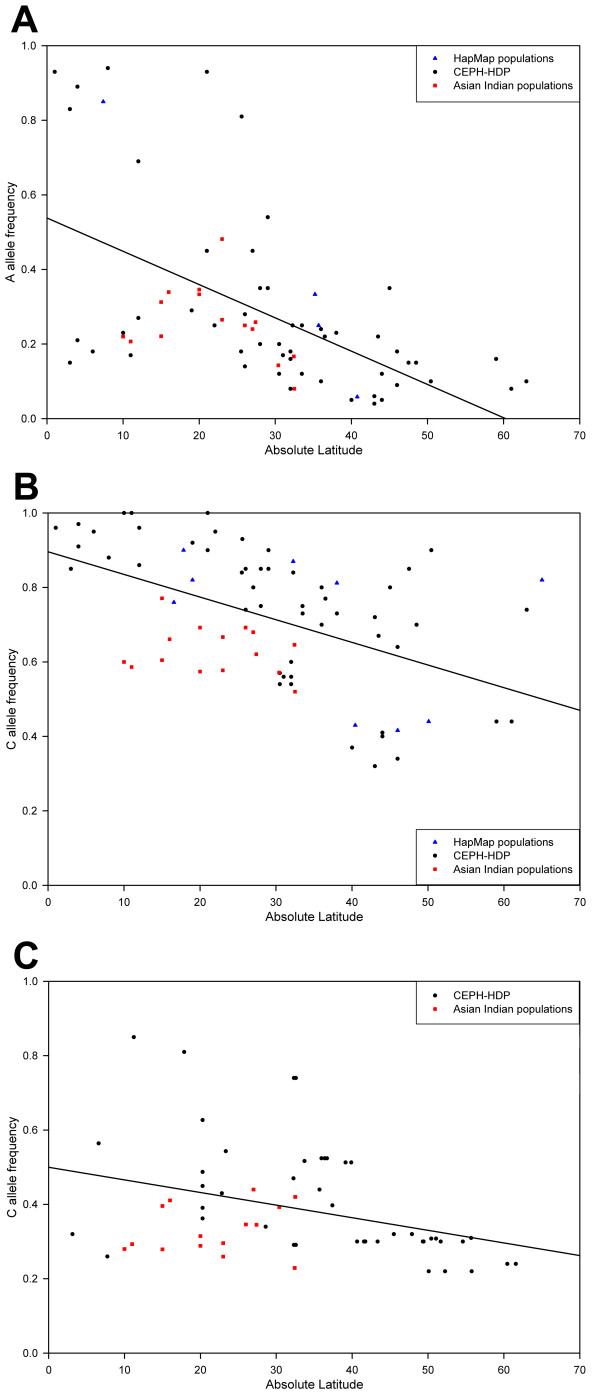
The correlation between minor allele frequency and latitude of (**A**) *CYP3A5 *g.6980G>A, (**B**) *AGT *g.802C>T, and (**C**) *GNB3 *g.4423C>T. Correlation coefficients for each graph can be found in Table 5.

The frequency of the *CYP3A5 *g.6980G>A [50,51], *AGT *g.802C>T [50], and *GNB3 *g.825C>T [52-54] polymorphisms have been reported to vary significantly between different populations/ethnicities in a cline that correlates with the latitudinal variation of the populations [50,55,56]. To investigate how this Indian cohort and its language groups fit into this correlation, the MAFs of the individual groups were plotted against absolute latitude together with the MAFs of the other known populations previously investigated with regard to the latitudinal cline (Figure 2; see Additional file 1, Tables S4–6). The MAFs of the Indian language groups for the *CYP3A5 *g.6980G>A SNP were found to follow the pattern of the other populations (Figure 2A). Accordingly, the correlation of allele frequency with latitude, as measured by ρ and r^2^, remained similarly high whether or not the Indian data were included (Table 5). A corresponding correlation was not found for the *AGT *g.802C>T SNP, for which the language groups were clustered largely below the other populations (Figure 2B). This resulted in an appreciable reduction in ρ and r^2 ^with the inclusion of the Indian data (Table 5), but the correlation remained significant at the 0.01 level. For the *GNB3 *g.825C>T SNP, the MAFs of the language groups largely cluster slightly below those of the other populations (Figure 2C). However, there was a substantial reduction of both ρ and r^2 ^when the Indian data were included (Table 5), reducing the significance of the correlation to the 0.05 level.

**Table 5:** Correlation coefficients of hypertension-associated polymorphism MAFs with latitude.

**Polymorphism**	**Population**	**Adjusted Spearman Rank Correlation Score**	**Adjusted r^2^**
*CYP3A5 *g.6980G>A	CEPH-HGDP	-0.621**	0.319
	CEPH-HGDP + HapMap	-0.620**	0.349
	CEPH-HGDP + HapMap + AIP	-0.603**	0.302

*AGT *g.802C>T	CEPH-HGDP	-0.718**	0.409
	CEPH-HGDP + Others	-0.693**	0.373
	CEPH-HGDP + Others + AIP	-0.518**	0.248

*GNB3 *g.825C>T	Others	-0.620**	0.278
	Others + AIP	-0.300*	0.101

* significant at 0.05, ** significant at 0.01

CEPH-HGDP = Centre d'Etude du Polymorphisme Humain-Human Genome Diversity Panel.

AIP = Asian Indian population.

Others = data from other populations that were not part of CEPH-HGDP or HapMap.

For sample sizes, please see Additional file 1, Tables S4, S5, S6, for *CYP3A5 *g.6980G>A, *AGT *g.802C>T, and *GNB3 *g.825C>T, respectively.

### Atherosclerosis

Whilst deviation away from the common 5-allele of the *ALOX5 *promoter microsatellite (ALOX5P) has been previously associated with an increased risk of atherosclerosis [57], allelic variation at the three *ALOX5 *coding SNPs (g.20C>T; g.8322G>A; g.50778G>A) has not. Haplotype analysis of ALOX5P and these three *ALOX5 *SNPs in this Indian cohort found that they were in strong LD, with a D' close to 1 (data not shown). However, their squared correlation (r^2^) was found to vary (Table 6). The 4-repeat allele of ALOX5P was found to be strongly correlated with the minor T-allele of the g.20C>T SNP, and the common 5-repeat allele was strongly correlated with the major C-allele of the g.20C>T SNP. The 3-repeat allele of the promoter repeat polymorphism was also found to be in moderate correlation with the major G-allele of the g.50778G>A SNP. In agreement with this correlation and with the allele frequencies, the most common haplotype of the ALOX5P promoter variant and the coding *ALOX5 *SNPs (g.20C>T; g.8322G>A; g.50778G>A) in the Indian population was 5CGG. The frequency of this haplotype was four times that of the second most common haplotype, which combined the 4-repeat allele of ALOX5P with the minor allele of the g.20C>T SNP and the major allele of the g.8322G>A and g.50778G>A SNPs (Table 7). Over half of the Indian cohort was homozygous for the 5CGG haplotype (Table 8) and a further third were heterozygous, of which 3.3% also possessed a second haplotype containing the 5-repeat allele of ALOX5P (Table 9). Two thirds of this cohort was heterozygous for the 5CGG and 4TGG haplotypes, and just over a fifth were heterozygous for the 5CGG and 6CGG haplotypes (Table 8). Three other haplotypes were found to be homozygous in individuals in this cohort but at a very low frequency; 4TGG, 6CGG, and 3CGG (Table 8).

**Table 6:** Squared correlation (r^2^) values between alleles of ALOX5P and the three coding *ALOX5 *SNPs.

**Allele**	**g.20C>T**	**g.8322G>A**	**g.50778G>A**
3	8.79 × 10^-5^	9.87 × 10^-5^	**0.253 [G]**
4	**0.949 [T]**	0.004	1.89 × 10^-5^
5	**0.674 [C]**	0.006	0.013
6	0.014	0.001	2.67 × 10^-4^
7	2.07 × 10^-4^	1.43 × 10^-5^	3.94 × 10^-6^
8	2.07 × 10^-4^	1.43 × 10^-5^	3.94 × 10^-6^

Significant r^2 ^values are shown in **bold**.

**Table 7:** Frequencies of the identified *ALOX5 *polymorphism haplotypes.

**Haplotype**	**Frequency**
**5CGG**	0.726
4TGG	0.179
6CGG	0.046
**5CAG**	0.017
4CGG	0.012
6TGG	0.010
3CGG	0.003
3CGA	0.003
**5CGA**	0.002
3TGG	0.001
**5TGG**	0.001
7CGG	0.001
8CGG	0.001

Polymorphisms are ordered ALOX5P→g.20C>T→g.8322G>A→g.50778G>A. Haplotypes containing common ALOX5P 5-repeat allele are shown in **bold**.

**Table 8:** Frequencies of the identified *ALOX5 *polymorphism diplotypes.

**Diplotype**	**Frequency**
**5CGG**/**5CGG**	0.531
4TGG/**5CGG**	0.253
**5CGG**/6CGG	0.086
4TGG/4TGG	0.051
**5CGG**/5CAG	0.031
4CGG/6TGG	0.016
3CGA/**5CGG**	0.005
6CGG/6CGG	0.003
4CGG/**5CGG**	0.003
4CGG/5CGA	0.003
**5CGG**/6TGG	0.003
3CGG/3CGG	0.002
3CGG/**5CGG**	0.002
3TGG/4TGG	0.002
4CGG/4TGG	0.002
4TGG/5CAG	0.002
**5CGG**/7CGG	0.002
**5CGG**/8CGG	0.002
**5CGG**/5TGG	0.002

Polymorphisms are ordered ALOX5P→g.20C>T→g.8322G>A→g.50778G>A.

Haplotype containing the common allele for all polymorphisms is shown in **bold**.

Haplotypes containing the common ALOX5P 5-repeat allele but not the common allele of all three SNPs are underlined.

**Table 9:** Frequencies of the *ALOX5 *diplotypes containing the common 5-repeat allele of ALOX5P.

**Diplotype**	**Frequency**
5CGG/5CGG	0.531
5CGG/5*	0.033
5CGG/*	0.356
5*/*	0.005
*/*	0.075

* indicates all other haplotypes.

### Prostate Cancer

The DG8S737 -1 allele and the A allele of rs1447295 were reported by Amundadottir *et al*. to be in strong linkage disequilibrium (LD) within the Icelandic population (D' = 0.85; r^2 ^= 0.52), the HapMap Caucasian American (D' = 0.72; r^2 ^= 0.29) and African (D' = 0.62; r^2 ^= 0.21) populations, and African Americans (D' = 0.48; r^2 ^= 0.12) [58]. Interestingly, haplotype analysis between the DG8S737 microsatellite and the rs1447295 SNP in this Indian cohort found that whilst the -1 microsatellite allele was in complete LD with the A-allele of rs1447295, the alleles were weakly correlated (Table 10). Rather, it was the rarer -4 allele (19 repeats) of DG8S737 that exhibited the greatest correlation with the A-allele of rs1447295 (Table 10).

**Table 10:** Linkage disequilibrium (D') and the squared correlation (r^2^) values between alleles of DG8S737 and rs1447295.

**Allele**	**D'**	**r^2^**
-10	0.551	0.005
-9	1.000	0.002
-8	1.000	3.88 × 10^-4^
-7	0.326	0.001
-6	0.160	0.002
-5	0.812	0.055
**-4**	**0.802**	**0.403 [A]**
-3	0.345	0.043
-2	0.417	0.004
-1	1.000	0.048
0	1.000	0.022
1	0.972	0.015
2	1.000	0.008
3	0.866	0.012
4	1.000	0.006
5	1.000	0.002
6	0.551	0.005
7	0.326	0.001

Allele -1 is equivalent to the prostate cancer risk-associated -8 allele reported by Amundadottir *et al*. that had 22 repeats [58].

In this Indian cohort, the haplotype containing the minor A-allele of the rs1447295 SNP and the -4 allele of DG8S737 was found to represent 7.1% of the haplotypes (Table 11). A further 1.4% carried the -4 allele of DG8S737 but the major C-allele of rs1447295, and 5.8% carried the minor A-allele of rs1447295 but a DG8S737 allele other than -4 (Table 11). The remaining 85.7% of haplotypes were found to contain the major C-allele of rs1447295 and a DG8S737 allele other than -4 (Table 11), which resulted in 73.4% of this Indian cohort being homozygous for these haplotypes (Table 12). We also found that 1.2% of this cohort was homozygous for the haplotype containing the -4 allele of DG8S737 and the minor A-allele of rs1448295, and a further 11.8% were heterozygous for this haplotype (Table 12). Of the latter 11.8%, a sixth had a second haplotype that contained the -4 allele of DG8S737 but not the minor A-allele of rs1448295, and just over half had a haplotype that contained the minor A-allele of rs1448295 but not the -4 allele of DG8S737. Interestingly, there were no haplotypes containing the -1 allele of DG8S737 and the minor A-allele of rs1447295, with the -1 allele found always associated with the C-allele of rs1447295. However, 6.6% of the individuals carried a single copy of both the -1 allele and the A-allele (data not shown).

**Table 11:** Haplotype frequencies for the -4 allele of the DG8S737 microsatellite and the alleles of rs1447295.

**Haplotype**	**Frequency**
-4/C	0.014
-4/A	0.071
*/C	0.857
*/A	0.058

* denotes all other DG8S737 alleles.

**Table 12:** Diplotype frequencies for the -4 allele of the DG8S737 microsatellite and the alleles of rs1447295.

**Diplotype**	**Frequency**
(*/C)/(*/C)	0.734
(*/C)/(*/A)	0.109
(*/C)/(-4/C)	0.026
(*/C)/(-4/A)	0.109
(*/A)/(-4/A)	0.007
(-4/C)/(-4/A)	0.002
(-4/A)/(-4/A)	0.012

* denotes all other DG8S737 alleles.

### Ability to taste PTC

The ability to taste PTC has been previously associated with the haplotypes formed by three SNPs in the *TAS2R38 *gene; g.144C>G, g.784C>T, and g.885G>A [59]. Haplotype analysis of these three SNPs found that the haplotype associated with an inability to taste PTC [59,60] was almost twice as prevalent in this Indian cohort (AVI) than the taster haplotype (PAV), with recombinants between these two haplotypes being extremely rare by comparison (Table 13). Almost half of the Indian cohort was found to be heterozygous for the taster/non-taster haplotypes AVI/PAV (Table 14). The most common homozygous haplotype was that of the non-taster AVI/AVI, which occurred almost four times as frequently as homozygosity for the taster haplotype PAV/PAV. Only 4.2% of the cohort possessed one of the taster or non-taster haplotypes and one of the recombinant haplotypes (Table 14). No individuals were found to possess only recombinant haplotypes.

**Table 13:** *TAS2R38 *haplotypes, their corresponding prototypes, and frequencies in the Asian Indian cohort.

**Haplotype**		
	
**Genotype**	**Prototype**	**Frequency**
GTA	AVI	0.630
CCG	**PAV**	0.345
GCG	AAV	0.014
GTG	AVV	0.006
CTG	PVV	0.003
CCA	PAI	0.001

Polymorphisms ordered as g.144C>G, g.784C>T, and g.885G>A

Taster amino acid haplotype (prototype) is shown in bold (**PAV**), and the non-taster haplotype is shown underlined (AVI).

**Table 14:** Individual diplotypes of the identified *TAS2R38 *polymorphisms and their corresponding frequencies in the Asian Indian cohort.

**Diplotype**		
	
**Genotype**	**Prototype**	**Frequency**
GTA/CCG	AVI/**PAV**	0.437
GTA/GTA	AVI/AVI	0.402
CCG/CCG	**PAV**/**PAV**	0.120
GCG/GTA	AAV/AVI	0.017
GTG/CCG	AVV/**PAV**	0.009
GCG/CCG	AAV/**PAV**	0.007
CTG/GTA	PVV/AVI	0.005
CCG/GTG	**PAV**/AVV	0.002
GTA/GCG	AVI/AAV	0.002

Polymorphisms ordered as g.144C>G, g.784C>T, and g.885G>A

Taster haplotype is shown in bold (**PAV**), and the non-taster haplotype is shown underlined (AVI).

**Figure 3:**
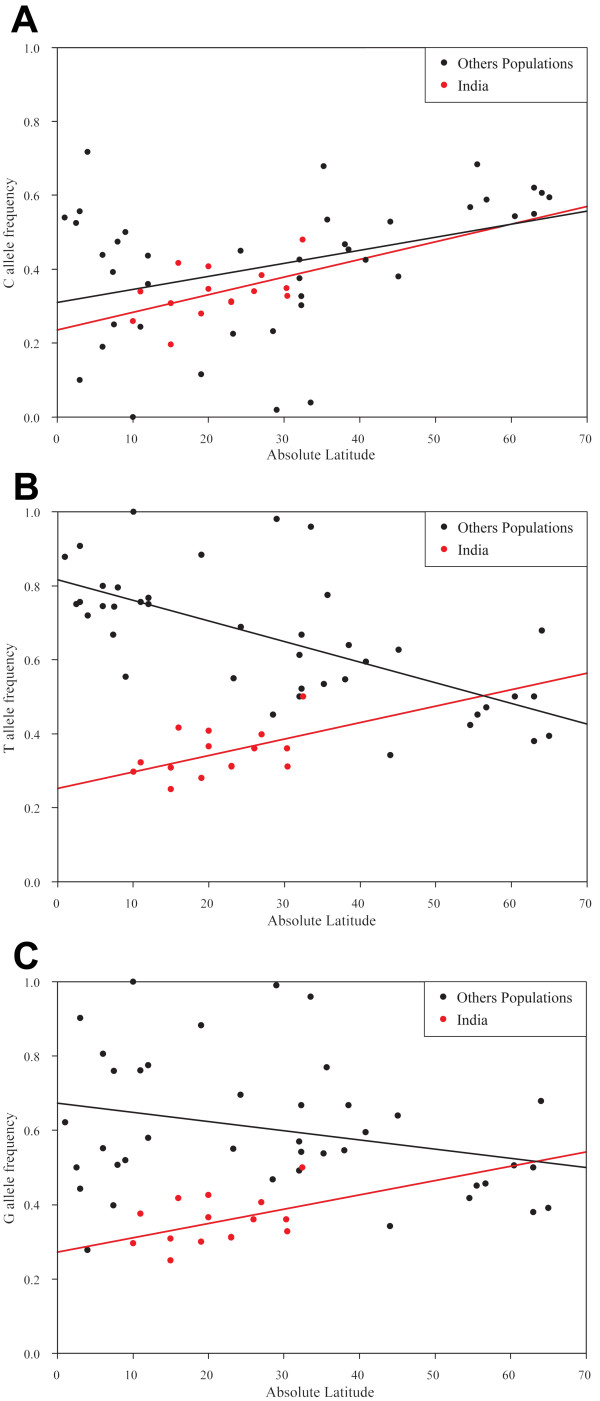
The correlation between latitude and minor allele frequency of the *TAS2R38 *SNPs (**A**) g.144G>C, (**B**) g.784C>T, and (**C**) g.885A>G. Correlation coefficients for each graph can be found in Table 15.

As observed above, the Kashmiri group had the highest MAFs for all three *TAS2R38 *SNPs and the Kannada group had the lowest (Table 4). Based on their relative frequencies, the Kashmiri would therefore be expected to have twice the number of tasters as the Kannada. The Kashmiri are one of the most northerly groups and the Kannada one of the most southerly (Figure 1), suggesting a possible latitudinal cline for the ability to taste PTC, with the greatest number of tasters in the northern groups and the least in the southern groups. This is supported by the MAF of the three SNPs in the other Indian language groups (Figure 3; Table 15). However, only the *TAS2R38 *g.144G>C SNP follows the same trend in both the Indian groups and in the other available world populations (Figure 3A; see Additional file 1, Table S18). For both the *TAS2R38 *g.784C>T and g.885A>G, the trend observed with the other world populations is the opposite of that observed with the Indian language groups (Figures 3B &3C, respectively; see Additional file 1, Tables S19 & S20, respectively). For example, the Indian groups display a decrease in MAF as the distance from the equator decreases, whereas other world populations demonstrate an increase in MAF as the distance from the equator decreases. The correlation between frequency and latitude was found to be the strongest for the *TAS2R38 *g.144G>C SNP, both when the Indian groups and the other world populations were considered separately and when they were combined (Table 15; Figure 3A &3B). The correlation was weakest for the *TAS2R38 *g.885A>G SNP, where it was still appreciable for the Indian groups, but less so for the other world populations (Table 15; Figure 3C).

**Table 15:** Correlation coefficients of the MAFs of the three *TAS2R38 *SNPs with latitude.

**Polymorphism**	**Population**	**Adjusted Spearman Rank Correlation Score**	**Adjusted r^2^**
g.144G>C	AIP	0.535*	0.186
	Others	0.386*	0.126
	Others + AIP	0.369**	0.154
g.784C>T	AIP	0.474	0.180
	Others	-0.670**	0.429
	Others + AIP	-0.305*	0.085
g.885A>G	AIP	0.433	0.118
	Others	-0.241	0.055
	Others + AIP	0.063	-0.017

* significant at 0.05, ** significant at 0.01

AIP = Asian Indian population

Others = data from non-Asian Indian populations

For sample sizes, please see Additional file 1, Tables S18, S19, §20, for g.144G>C, g.784C>T, and g.885A>G, respectively.

## Discussion

We have reported here the frequencies of common polymorphisms associated with an increased risk of several diseases or phenotypes, within a cohort of 576 Indian-born Asian Indians. It is important to note that because the study participants were born in India, we refer to the individuals and populations as being "Asian Indian" or "Indian"; however, it is important to note that because the Asian Indian individuals were sampled in the United States, and therefore do not represent a random sample from India or all of the different castes, tribes, or language groups, some biases may be introduced when extrapolating the results to India as a whole [10]. It is also important to note that because Hindi is spoken by 43 percent of the Indian population, primarily in the states of Northern India, heterogeneity will be present between its various constituent population groups. Our Hindi language group is a sample of individuals who emigrated from a restricted region of India, and therefore biases may be introduced when extrapolating their results to Hindi speakers as a whole.

All polymorphisms, with the exception of *ALOX5 *g.20C>T, were found to be in HWE. The deviation away from HWE for the *ALOX5 *g.20C>T SNP is most likely due to chance, but given its low frequency we cannot exclude the possibility of genetic drift, differing selection between the groups, or the small sample size of all but one (Gujarati) of our language groups. Genotyping error could be another possible explanation, but the extremely high accuracy of the TaqMan genotyping assay [61] and the 100% concordance of replicated genotypes makes this explanation unlikely.

No significant population subdivision was identified by F_ST _between the different Indian language groups (Tables 2, 3, &4). However, variation in the MAF of some of the polymorphisms between the different language groups was observed. Furthermore, the *ALOX5 *g.8322G>A and g.50778G>A SNPs, as well as the *PTPN22 *g.36677C>T SNP, were found to be present in some but not all of the Indian language groups investigated here. There was no discernible pattern to presence/absence of the minor allele of these SNPs with regard to the location of these groups within India (Figure 1), and it is possible that their presence or absence is the result of either the different origins of the language groups during the peopling of India and strengthened by their practice of endogamy [62,63], or admixture introduced by the differential migration of non-Indian populations into regions of India during the course of its history [64,65]. With the exception of the Gujaratis, the small sample size of our individual language groups could also be responsible for this observation as we may be under powered to detect the minor allele in these groups. However, the absence of the *ALOX5 *g.8322G>A, *ALOX5 *g.50778G>A, and *PTPN22 *g.36677C>T SNPs in the Gujarati language group, for which we have around seven times the number of individuals of the other groups, would suggest that their absence in the other groups may not necessarily be due to a lack of a sufficient sample size for their detection. If this observed variation in the MAF of some of the polymorphisms between the different language groups is confirmed, then this could impact the design of future case-control studies in India as it would suggest that the inclusion of a disproportionate number of individuals from a particular language group in either the case or control groups could lead to missed or spurious associations.

Interestingly, the MAF of the atherosclerosis (ALOX5P, *ALOX5 *g.20C>T, g.8322G>A, and g.50778G>A), type-1 and type-2 diabetes (*CAPN10 *g.4834T>C, *TCF7L2 *g.98386G>T, and *PTPN22 *g.36677C>T), and Hirschsprung disease (*RET *g.9349G>A) associated polymorphisms were found to be similar to those of European populations. Similarly, the frequencies of variants associated with AMD (*CFH *g.37989T>C and *LOC388715 *g.205G>T) and prostate cancer (DG8S737 and rs1447295 were similar to those of African populations. However, none showed a high similarity to the populations of Eastern Asia. This could reflect a greater degree of non-Asian compared to Asian gene flow into India during the course of its history; however, if higher caste is correlated with a European or western Asian component of ancestry, this could be a reflection of our sampling of Asian Indian immigrants in the United States that are likely biased towards the higher castes.

The frequency of the *CYP3A5 *g.6980G>A, *AGT *g.802C>T, and *GNB3 *g.825C>T polymorphisms have been reported to vary significantly between different populations/ethnicities in a cline that correlates with variation in the latitudinal position of the populations [50,55,56]. We have shown that whilst the MAF of the *CYP3A5 *g.6980G>A SNP in the Indian groups agrees with the trend identified in other world populations, the inclusion of the Indian data reduces the previously reported correlation between the frequency of the *AGT *g.802C>T and *GNB3 *g.4423C>T SNPs and latitude. The association of the MAF of these three SNPs with changes in latitude, purportedly due to their effects on sodium homeostasis, suggests that the relevant selective pressures within India are not themselves strongly correlated with latitude.

An association between changes away from the common allele (5 repeats) in a repeat-polymorphism (ALOX5P) comprised of Sp1 binding sites in the promoter region of *ALOX5 *and atherosclerosis has been previously reported [57]. Analysis of ALOX5P in the Indian cohort identified six alleles; 56.4% of individuals were homozygous for the common allele of five Sp1-binding site repeats, and 92.5% of individuals had at least one copy of the common allele. The remaining 7.5% of individuals had two variant alleles, which is much lower than the incidence of CAD in the Indian population [16,66]. This is higher than the 5.96% reported for a cohort of individuals of mixed-ethnicity, who reported 94.0% of individuals with at least one copy of the common allele [57]. The same study also reported that variant alleles were more common in blacks (24.0%) and Asians or Pacific Islanders (19.4%) than in Hispanic (3.6%) and non-Hispanic whites (3.1%). The frequency of variants in this Indian cohort would suggest that their frequency is closer to Caucasians than Asians.

Three coding SNPs have also been identified in the *ALOX5 *gene (g.20C>T, g.8322G>A, and g.50778G>A) and these appear to be in partial or complete LD with the alleles of ALOX5P (HA; unpublished data). Haplotype analysis of the four *ALOX5 *polymorphisms in this Indian cohort found that there is LD between three alleles of ALOX5P – the major and minor alleles of the g.20C>T SNP (5 and 4 repeats, respectively), and the major allele of g.50778G>A SNP (3 repeats). Haplotypes containing variant alleles of ALOX5P are present in almost half of the cohort, with 8.1% of the cohort homozygous for haplotypes carrying a variant allele.

Analysis of the type-2 diabetes-associated SNPs *CAPN10 *g.4834T>C and *TCF7L2 *g.98386G>T, found their MAFs (23.1% and 24.8%, respectively) to be almost twice as common as type-2 diabetes in the Indian population (11.6%) [22]. Two recent studies have reported that the *TCF7L2 *g.98386G>T variant is strongly associated with type-2 diabetes risk in a Asian Indians [67,68], and our frequency of this variant (24.8%) in this Indian cohort is close to that reported for control subjects (22%) [68]. The *PTPN22 *g.36677C>T SNP has a low MAF (0.009) in our Indian cohort, comparable to that previously reported (0.012) [69] and almost one hundred fold higher than the reported prevalence of type-1 diabetes in the Indian population (0.011–0.026%) [30,31]. The minor allele of *PTPN22 *g.36677C>T SNP is absent in other Asian populations, but present in those of European origin (see Additional file 1, Table S9), suggesting that its presence in Asian Indians is through the introduction of admixture by migrating individuals of European origin into India during the course of its history.

Interestingly, the most common allele of the DG8S737 microsatellite in this Indian cohort has been associated with an increased risk of prostate cancer in other populations (-1 allele) [58]. However, whilst this allele has been reported to be in strong LD with the minor A-allele of SNP rs1447295 in other populations, we have found that a different allele (-4 allele) is in strong LD with the minor A-allele of SNP rs1447295 in this Indian cohort. The frequency of these polymorphisms is also still far higher than the frequency of prostate cancer in the Asian Indian population (10 per 100,000) [28,29]. Further studies on Asian Indian prostate cancer cases may allow for the true causative variant to be identified.

The moderate frequency of the Hirschsprung disease-associated minor allele of *RET *g.9349G>A in the Indian cohort is in contrast with the relatively low frequency of the disease in India (2.8 per 10,000 births) [25]. However, the MAF of the *CFH *g.37989T>C and *LOC387715 *g.205G>T SNPs in this Indian cohort is in agreement with the low frequency of AMD in the Indian population [27].

Previous investigation suggests that the PAV "taster" haplotype is dominant over the other recessive haplotypes including the "non-taster" AVI haplotype as PAV/AVI individuals were largely able to taste PTC [59,70]. We would therefore expect, based upon the frequencies of the different haplotypes in this Indian cohort, that 57.4% of the population would be able to taste PTC (Table 16). This value is very close to the percentage of the population identified as able to taste PTC in previously reported studies: Gujarat (57.1%), Kerala (51.6%), Maharashtra (54.9%, 57.6% and 63.7%), Tamil Nadu (59.2%), and Uttar Pradesh (69.1%) [71-75]. These data would suggest that the lower than expected number of Indians able to taste PTC when compared with the average of 75% in worldwide populations [76,77] is due to an increased presence of the non-taster haplotype in this population. In agreement with this notion, we observed that the frequency of the haplotype of the three SNPs in the *TAS2R38 *gene associated with the ability to taste PTC in this Indian cohort is almost 20% lower than other world populations.

**Table 16:** Frequencies of taster and non-taster associated diplotypes in the Asian Indian cohort.

**Diplotype**		
	
**Genotype**	**Prototype**	**Frequency**
CCG/CCG	**PAV**/**PAV**	0.120
CCG/*	**PAV**/*	0.017
GTA/CCG	AVI/**PAV**	0.437
GTA/*	AVI/*	0.024
GTA/GTA	AVI/AVI	0.402

Polymorphisms ordered as g.144C>G, g.784C>T, and g.885G>A

Taster haplotype is shown in bold (**PAV**), and the non-taster haplotype is shown underlined (AVI).

Interestingly, the MAFs of the three *TAS2R38 *SNPs were found to vary in the Indian groups in a cline that correlated with the latitudinal variation of the groups. The MAFs of all three SNPs were higher in the more northerly Indian groups than in the more southerly groups, suggesting a greater frequency of tasters in the former than the latter. However, this trend was only observed in the other world populations for the *TAS2R38 *g.144G>C SNP, with the trend reversed for both the *TAS2R38 *g.784C>T and g.885A>G SNPs.

One possible explanation for the differences between the trends in the Indian groups and other world populations could be the differential migration of non-Indian individuals into different parts of India during the course of its history. This could result in variation in the degree and source of the admixture introduced into the different language groups such that their frequencies deviate from the trends of other populations. There could also be selective pressures that are unique to, more important in, or different in action in, India than the other world populations, and that also share a strong correlation with latitude.

It has been previously reported that balancing selection is acting on the *TAS2R38 *receptor gene, possibly due to the different taster and non-taster alleles encoding receptors that recognize different, but equally important, ligands [60,77]. The latitudinal cline we have identified in our Indian language groups with regards to the taster/non-taster allele frequencies could therefore be the result of the strength of the selective pressures exerted by these ligands affecting individuals in the north and south of India differently, but for reasons that are distinct from other world populations. Another possible selective pressure relates to bitter taste perception and the acceptance of foods containing increasing amounts of spice [78]. This is of particular interest given that patterns of spice use have been reported to both vary between populations and regions, and also to have a significant latitude correlation, where more spices tend to be used in warmer climates purportedly due to their powerful antimicrobial action [79]. The climate differences between the more arid south of India and the more mountainous north, and the incorporation of a lot of spices in Indian cooking, makes this a possible explanation for our latitudinal correlation. It has also been proposed that the local adaptation in human bitter taste receptor genes is common and has been driven by the fitness advantages of avoiding toxic, bitter compounds found in plants [80]. If the composition and abundance of ligands to which the different taster and non-taster alleles of the *TAS2R38 *gene vary geographically in India in a cline associated with latitude, this could also explain our observed latitudinal correlation.

The MAF for the *SLC24A5 *g.13242G>A polymorphism (0.114) in the Indian population is closer to that of Caucasian European populations (0.000–0.020) than to that of East Asians (0.979–0.989) and Africans (0.730–0.980) (see Additional file 1, Table S14). This is surprising given that, as a whole, people of Indian origin have darker skin tone compared to Europeans. The East Asian and African populations share a similarly high frequency of the minor allele of the *SLC24A5 *g.13242G>A SNP, which in itself is surprising as East Asians are typically of much lighter skin than Africans. It is also interesting that the t-test suggested a greater similarity in MAF between Indians and those of populations in the Americas (Puerto Rican, Mexican, and Amerindian), who are of a similar skin tone (Table 4). The presence of the minor allele of the *SLC24A5 *g.13242G>A SNP cannot therefore, completely explain Indian skin color, making the investigation of polymorphisms in additional genes implicated in skin pigmentation [81] essential to understanding the intricacies of their skin color.

We have found that, with the exception of the *TAS2R38 *haplotypes associated with the ability to taste PTC, the frequency of the disease/trait-associated minor alleles of the polymorphisms investigated in this Indian cohort did not correlate with the reported disease/trait prevalence in the Indian population. This is unsurprising given that, with the exception of the ability to taste PTC, all are known to be complex diseases/traits that are the result of the combined effect of variants at multiple genetic loci and environmental factors. Variation in genetic backgrounds and environmental heterogeneity between populations will likely have a modifying effect on the correlation of the allele frequency of polymorphisms with disease/trait prevalence as their contribution to the presentation of the disease/trait varies. The degree of complexity between these disease/traits will also vary, as some, such as CAD, are known to be highly complex whereas others, such as AMD, may be less complex. One might therefore not expect the correlation of factors associated with CAD to be as strong as those associated with AMD, and therefore the lack of a strong correlation between many of these polymorphisms and their respective complex disease/trait prevalence most likely indicates that the environmental and/or other genetic factors make a stronger contribution to the presentation of the disease/trait in Indians than do these variants alone. Interestingly, the two polymorphisms associated with AMD (*CFH *g.37989T>C &*LOC387715 *g.205G>T), whilst not showing strong correlation between their disease-associated MAF and reported disease prevalence in the Indian population, did show a frequency that correlated with the MAF of these SNPs in other populations and the prevalence of AMD in those populations, suggesting that they may share similar risk factors.

## Conclusion

The Asian Indian population represents a large population within which many complex-trait disorders are found at a high frequency. The high prevalence of endogamy and relatively low admixture present in this population distinguishes it from most other populations presently used in genetic studies as it represents a distinct genetic background that is largely unaffected by outside admixture. In some cases, such as coronary artery disease, Asian Indians exhibit unique phenotypic characteristics that distinguish them from the other populations, suggesting that unique causative factors underlie this and possibly other related diseases. Our results suggest that caution should be used when treating the Asian Indian population as a single population in genetic studies using SNPs. The overall difference in minor allele frequency of the polymorphisms investigated was small between the different language groups, but some inter-group variation was observed. However, caution is warranted in interpreting our results as whilst our Indian cohort is likely to be reasonably representative of first-generation individuals of Asian Indian descent currently residing in the United States, individuals from relatively mobile populations and those of higher caste and socioeconomic status are likely overrepresented within this cohort. Such individuals likely do not provide a random sample of the source populations in India and therefore if variables such as caste and socioeconomic status do have a significant effect on genotype frequency in India, this will not be reflected in these results. Additionally, if higher caste is correlated with a European or western Asian component of ancestry, a sample in the United States may be biased towards finding a greater similarity of populations from India to those of the Europe/Middle East rather than to those of East Asia [10], as we have discovered for many of the polymorphisms reported here. We must also use caution when interpreting the variation at the *PTPN22 *g.1825C>T and *ALOX5 *g.50778G>A loci between the 15 language groups, as the small sample size for each group, with the exception of the Gujarati, is a limiting factor in our ability to detect polymorphisms with a low minor allele frequency. However, our results do support the inclusion of the Indian population in modern genetic association studies as these data suggest that Asian Indians likely exhibit unique genotype as well as phenotype characteristics that may yield new insight into the underlying causes of diseases that are not available in other populations.

## Methods

### Study population

The Asian Indian cohort used in this study was as previously described [10]. Our sample consisted of an expanded population of 576 individuals of Indian-born Asian Indians living in the United States who have largely emigrated from Indian cities. This population was designed so that when subdividing study participants by their self-reported primary spoken Indian language, 15 languages (Assamese, Bengali, Gujarati, Hindi, Kannada, Kashmiri, Konkani, Malayalam, Marathi, Marwari, Oriya, Parsi, Punjabi, Tamil, and Telugu), each having a relatively localized distribution within India (Figure 1), would be well-represented. The number of Gujarati individuals sampled (181) was larger than the other language groups (ca. 25–30) to examine rare minor allele frequencies. Three other language groups (Dogri, Sindhi, and Tulu) were present in the population but not at a significant number to be analyzed independently, but these were retained for whole cohort analysis. A sample size of at least 24 per population has been found to be reasonably suitable for population structure analysis [82,83], as well as for microsatellite studies of linkage disequilibrium [84], suggesting that it is suitable for this study.

### Marker genotyping

All single nucleotide polymorphisms (SNPs) were interrogated using TaqMan allelic discrimination assays (Applied Biosystems, Foster City, CA) following the manufacturer's recommended protocol for a 5 μl reaction volume and 2ng of dried DNA using an Applied Biosystems (Foster City, CA) 7900HT fast real-time PCR system. ABgene (Rochester, New York) ABsolute™ QPCR ROX (500 nM) mix was used for all assays. Pre-designed assays were available for all SNPs except *CFH *g.37989T>C (Probe: 5'-TTTCTTCCAT [G/A]ATTTTG-3'; Forward primer: 5'-CTTTATTTATTTATCATTGTTATGGTCCTTAGGAAAATGTTATTT-3'; Reverse primer: 5'-GGCAGGCAACGTCTATAGATTTACC-3'), *ALOX5 *g.20C>T (Probe: 5'-TGGCCAC [G/A]GTGACC-3'; Forward primer: 5'-CGCCATGCCCTCCTACAC-3'; Reverse primer: 5'-AGTGCCGGCGAACCA-3'), *ALOX5 *g.8322G>A (Probe: 5'-CTGAAGAC[G/A]CCCCACG-3'; Forward primer: 5'-TGAATGACGACTGGTACCTGAAGTA-3'; Reverse primer: 5'-GGTGATCCAGCGGTAGCA-3'), and *ALOX5 *g.50778G>A (Probe: 5'-CTGCCC [G/A]AGAAGC-3'; Forward primer: 5'-AGACCTGATGTTTGGCTACCAGTT-3'; Reverse primer: 5'-CGCTCCAGGCTGCACTCTA-3'), for which custom assays were designed using the Applied Biosystems (Foster City, CA) assay-by-design service. All genotypes were determined using the AutoCall clustering algorithm of the Sequence Detection Systems (SDS) software version 2.1 (Applied Biosystems, Foster City, CA) with a quality value threshold of 95. Genotypes that could not be called using this algorithm and threshold were repeated along with a small number of successfully genotyped samples to validate their genotypes and confirm assay performance in the repeated data. No switches in genotypes were observed with these replicated samples.

Microsatellite markers were genotyped using fluorescent primers on an ABI3100 genetic analyzer (Applied Biosystems, Foster City, CA) and analyzed using Genotyper version 3.7 (Applied Biosystems, Foster City, CA). Primers for DG8S737 were as previously described [58] and PCR was performed using a Biometra (Goettingen, Germany) T-Gradient thermal cycler and the following cycle parameters: 95°C 5 mins followed by 35 cycles of 95°C for 45 sec, 60°C for 45 sec, 72°C for 45 sec, and a final hold at 72°C 10 mins before holding at 4°C. New England Biolabs (Ipswich, MA) Taq DNA polymerase (1U) and manufacturer supplied 10× buffer (including 1.5 mM MgCl_2_) was used along with 4 pmol of each primer, 30 μmol of each dNTP (Invitrogen, Carlsbad, CA), and 30 ng DNA in a 15 μl reaction volume. Alleles of DG8S737 were assigned based upon the change in the number of repeats away from that of the reference sequence in the NCBI database (23 AC dinucleotide repeats; Acc. # NT_008046.15: 148848–149016).

Primers for the *ALOX5 *promoter microsatellite (ALOX5P) were as previously described [85] but with the inclusion of the GTGTCTT pig-tail [86] and PCR was performed in an Applied Biosystems (Foster City, CA) GeneAmp 9700 thermal cycler using the following cycle parameters: 95°C 12 mins followed by 10 cycles of 94°C for 1 min, 68°C for 2 min, followed by 25 cycles of 94°C for 30 sec, 60°C for 30 sec, 72°C for 45 sec, and a final hold at 72°C 5 mins before holding at 4°C. Applied Biosystems (Foster City, CA) AmpliTaq Gold DNA polymerase (1.2U) and manufacturer supplied 10× buffer was used along with 1.5 mM MgCl_2_, 2% DMSO, 400 nmol of each primer, 30 μmol of dATP, dUTP, and dCTP (Invitrogen, Carlsbad, CA), 62.5 μmol 7-deaza-dGTP (New England Biolabs, Ipswich, MA), and 30 ng DNA in a 25 μl reaction volume. Labels for the alleles of ALOX5P were assigned based upon the number of hexanucleotide Sp1-binding sites that were present.

### Haplotype analysis

Haplotype phase was estimated for the *TAS2R38 *g.144G>C, g.784C>T, and g.885A>G SNPs using *fastPHASE *version 1.1.4 [87] using the following settings; 50 random starts of the EM algorithm, 50 iterations of the EM algorithm, 200 haplotypes sampled from the "posterior" distribution obtained from a particular random start of the EM algorithm, and haplotype frequencies estimated by Monte Carlo methods by sampling from the observed genotypes 10,000 times per diploid individual. As *fastPHASE *is unable to analyze microsatellite data, PHASE version 2.1 [88,89] was used to estimate haplotype phase for the ALOX5P and the three coding SNPs (g.20C>T, g.8322G>A, and g.50778G>A) and also for DG8S737 and rs1447295 using the following settings; a burn-in of 25,000 iterations followed by 10,000 main iterations with a thinning interval of 100 iterations. The final run used 10 times this number of burn-in and main iterations. Haplotypes were constructed using the hybrid haplotype reconstruction model that uses a model that ignores the decay of LD with distance [88] for preliminary computations, and a different model that makes explicit allowance for the decay of LD with distance [90] for the final computations.

### Statistical analysis

All statistics were calculated in R version 2.3.1 [91] unless otherwise stated. To test that all SNPs are in equilibrium within the Asian Indian cohort, a Hardy-Weinberg equilibrium (HWE) constant was calculated for each polymorphism using the *HWE.test *function in the *genetics *R-package [92]. A Pearson's χ^2 ^test with simulated p-value (based on 10000 iterations) was calculated for all HWE statistics as this avoids the reliance on the assumptions of the underlying Chi-square approximation, which is particularly important when some allele pairs have small counts [92]. An HWE score of 0 equates to the polymorphism being in complete Hardy-Weinberg equilibrium, and a score of 1 equates to the polymorphism being in complete Hardy-Weinberg disequilibrium. A standard error (SE) for the estimated allele frequency for each SNP was calculated by using the formula: SE = s/√n, where s is the estimated standard deviation and n is the size of the sample.

Weir and Cockerham F_ST _test statistics [93] were calculated for all SNP data as previously described [94] using R to measure the effect of population subdivision and the overall genetic divergence among these groups, where an F_ST _of 0 means no genetic divergence, and 1 means extreme genetic divergence, between the groups, and an F_ST _of up to 0.05 represents a relatively small level of genetic differentiation. Weir and Cockerham F_ST _test statistics for the microsatellite data were calculated using FSTAT version 2.9.3.2 [95]. To estimate the significance of these F_ST _values, a probability value (p) was calculated for each F_ST _value from a χ^2 ^distribution using the *pchisq *function in the *Stats *R-package using 14 degrees of freedom (number of Indian language groups minus 1) [96]. As it is possible for the unbiased estimate of F_ST _used here to assume negative values, which does not have a biological interpretation, we set negative values of F_ST _to 0 [94].

As it was unrealistic to assume that the variance within the Asian Indian cohort is equal to that of other populations, a Welch modified two-sample t-test with unequal variances was calculated between the minor allele frequency in the Indian cohort and those previously reported for other populations. This was performed using the *t.test *function in the *Stats *R-package with a 95% confidence interval to test whether or not the minor allele frequency in the Asian Indian cohort were significantly different from those of other populations. To give as accurate a representation of the MAF in the Asian Indian cohort, the MAF of each group was included in this analysis. Data on other populations was obtained from prior publications and the National Center for Biotechnology Information's (NCBI) dbSNP database [97,98]. These populations were grouped into the following geographical regions for comparison: Africa, Asia (including Central, Southern, Eastern, and South-East Asian populations), Americas (native populations of South and Central America, and native populations of North America), Europe (including non-native European American populations, and Russia), Oceania (Australia, New Zealand, and the Pacific islands), except for the analysis of *Cyp3A5 *g.6980G>A and *AGT *g.802C>T where there were sufficient Asian populations to allow Asia to be split into Central/South Asia and East Asia (East and South-Eastern Asia), and there were also sufficient populations to include a Middle East.

To test whether the polymorphisms associated with atherosclerosis (ALOX5P and the three *ALOX5 *coding-SNPs g.20C>T, g.8322G>A, and g.50778G>A), ability to taste PTC (the three *TAS2R38 *coding-SNPs g.144G>C, g.784C>T, and g.885A>G), and prostate cancer (the DG8S737 microsatellite and rs1447295 SNP), were in linkage disequilibrium, the linkage disequilibrium measure (D') and the squared correlation measure of linkage disequilibrium (r^2^) were calculated between the respective polymorphisms using the java based linkage disequilibrium plotter JLIN [99]. For the ALOX5P and DG8S737 microsatellite markers, each allele was tested individually with all other alleles collapsed into a single "super-allele".

Adjusted Spearman's rank correlation coefficients and squared correlation coefficients (r^2^) for the association of the *CYP3A5 *g.6980G>A, *AGT *g.802C>T, and *GNB3 *g.825C>T SNPs, and the *TAS2R38 *g.144G>C, g.784C>T, and g.885A>G SNPs, with absolute latitude were calculated using SPSS version 15 (SPSS Inc.; Chicago, IL). Graphs of their MAF against absolute latitude (Figures 2 &3) were plotted using SigmaPlot version 9 (Systat Software, Inc.; San Jose, CA).

## Abbreviations

Age-related macular degeneration (AMD); Coronary artery disease (CAD); Hardy-Weinberg equilibrium (HWE); Linkage disequilibrium (LD); Minor allele frequency (MAF); Phenylthiocarbamide (PTC).

## Competing interests

The author(s) declare that they have no competing interests.

## Authors' contributions

TJP and PIP conceived the study. TJP and NM performed the genotyping. TJP performed the data analysis with assistance from DVC AD and DW assisted with the analysis of the *CYP3A5 *and *AGT *polymorphisms, and HA assisted with the genotyping of the *ALOX5 *polymorphisms. The paper was written primarily by TJP and PIP, with assistance from DVC, HA, and AD.

## Supplementary Material

Additional file 1 Supplementary Tables 4–20. Allele frequencies of the 17 SNPs in other worldwide populations. Click here for file

Additional file 2 Supplementary Tables 1–3. Genotype frequencies of the 17 SNPs (Table S1), and the ALOX5P (Table S2) and DG8S737 (Table S3) microsatellites. Click here for file
