# Supplementary material for: Prevalence of common disease-associated variants in Asian Indians
Source: BMC Genet. 2008 Feb 4;9:13. doi: 10.1186/1471-2156-9-13 (PMC2267478; doi:10.1186/1471-2156-9-13)
Supplement: Additional file 1 — Supplementary Tables 4–20. Allele frequencies of the 17 SNPs in other worldwide populations. [file 1471-2156-9-13-S1.pdf]

Supplementary Table 4. Frequencies of the *Cyp3A5* g.6980G>A minor allele in other worldwide populations.

Population	Region	Latitude	N	MAF (A)	Reference
YRI (Ibadan, Nigeria)	Africa	7.38	60	0.850	[1]
CEPH - Nigeria (Yoruba)	Africa	8.00	25	0.060	[2]
CEPH - Congo (Mbuti Pygmies)	Africa	1.00	15	0.070	[2]
CEPH - Namibia (San)	Africa	-21.00	7	0.070	[2]
CEPH - Central African Republic (Biaka Pygmies)	Africa	4.00	33	0.110	[2]
CEPH - Kenya (Bantu N.E.)	Africa	-3.00	12	0.170	[2]
CEPH - S. Africa (Bantu S.E./S.W.)	Africa	-25.57	8	0.190	[2]
CEPH - Senegal (Mandenka)	Africa	12.00	24	0.310	[2]
CEPH - Mexico (Maya)	Americas	19.00	24	0.710	[2]
CEPH - Mexico (Pima)	Americas	29.00	25	0.460	[2]
CEPH - Brazil (Karitiana)	Americas	-10.00	24	0.770	[2]
CEPH - Brazil (Surui)	Americas	-11.00	21	0.830	[2]
CEPH - Colombia	Americas	3.00	13	0.850	[2]
CEPH - China (Uygur)	Central/South Asia	44.00	10	0.950	[2]
CEPH - Pakistan (Hazara)	Central/South Asia	33.50	25	0.750	[2]
CEPH - Pakistan (Kalash)	Central/South Asia	35.99	25	0.760	[2]
CEPH - Pakistan (Makrani)	Central/South Asia	26.00	25	0.860	[2]
CEPH - Pakistan (Brahui)	Central/South Asia	30.50	25	0.880	[2]
CEPH - Pakistan (Pathan)	Central/South Asia	33.49	25	0.880	[2]
CEPH - Pakistan (Burusho)	Central/South Asia	36.50	25	0.780	[2]
CEPH - Pakistan (Balochi)	Central/South Asia	30.50	25	0.800	[2]
CEPH - Pakistan (Sindhi)	Central/South Asia	25.49	25	0.820	[2]
CHB (Beijing, China)	East Asia	35.21	45	0.333	[1]
JPT (Tokyo, Japan)	East Asia	35.68	44	0.250	[1]
CEPH - China (Dai)	East Asia	21.00	10	0.550	[2]
CEPH - China (She)	East Asia	27.00	10	0.550	[2]
CEPH - China (Miaozu)	East Asia	28.00	10	0.650	[2]
CEPH - China (Tujia)	East Asia	29.00	10	0.650	[2]
CEPH - China (Mongolia)	East Asia	45.00	10	0.650	[2]
CEPH - China (Lahu)	East Asia	22.00	10	0.750	[2]
CEPH - China (Han)	East Asia	32.27	44	0.750	[2]
CEPH - China (Naxi)	East Asia	26.00	9	0.720	[2]
CEPH - China (Xibo)	East Asia	43.50	9	0.780	[2]
CEPH - China (Yizu)	East Asia	28.00	10	0.800	[2]
CEPH - China (Hezhen)	East Asia	47.50	10	0.850	[2]
CEPH - China (Daur)	East Asia	48.50	10	0.850	[2]
CEPH - China (Tu)	East Asia	36.00	10	0.900	[2]
CEPH - China (Orogen)	East Asia	50.43	10	0.900	[2]
CEPH - Cambodia	East Asia	12.00	11	0.730	[2]
CEPH - Japan	East Asia	38.00	31	0.770	[2]
CEPH - Siberia (Yakut)	East Asia	62.98	25	0.900	[2]
CEU (Utah, USA)	Europe	40.76	60	0.058	[1]
CEPH - Orkney Islands (Orcadian)	Europe	59.00	16	0.840	[2]
CEPH - Russia Caucasus (Adygei)	Europe	44.00	17	0.880	[2]
CEPH - France	Europe	46.00	29	0.910	[2]

CEPH - Russian	Europe	61.00	25	0.920	[2]
CEPH - Italy (Bergamo)	Europe	46.00	14	0.820	[2]
CEPH - Italy (Tuscan)	Europe	43.00	8	0.940	[2]
CEPH - Italy (Sardinian)	Europe	40.00	28	0.950	[2]
CEPH - France (Basque)	Europe	43.00	24	0.960	[2]
CEPH - Israel (Palestinian)	Middle East	32.00	51	0.820	[2]
CEPH - Israel (Bedouin)	Middle East	31.00	48	0.830	[2]
CEPH - Algeria (Mozabite)	Middle East	32.00	29	0.840	[2]
CEPH - Israel (Druze)	Middle East	32.00	48	0.920	[2]
CEPH - Melanesian (Bougainville)	Oceania	-6.00	22	0.820	[2]
CEPH - New Guinea (Papuan)	Oceania	-4.00	17	0.790	[2]

Supplementary Table 5. Frequencies of the *AGT* g.802C>T minor allele in other worldwide populations.

Population	Region	Latitude	N	MAF (T)	Reference
Zimbabwe (Harare)	Africa	-17.82	40	0.100	[3]
CEPH - Nigeria (Yoruba)	Africa	8.00	20	0.120	[2]
CEPH - Congo (Mbuti Pygmies)	Africa	1.00	13	0.040	[2]
CEPH – Namibia (San)	Africa	-21.00	7	0.000	[2]
CEPH - Central African Republic (Biaka Pygmies)	Africa	4.00	31	0.030	[2]
CEPH - Kenya (Bantu N.E.)	Africa	-3.00	10	0.150	[2]
CEPH - S. Africa (Bantu S.E./S.W.)	Africa	-25.57	7	0.070	[2]
CEPH - Senegal (Mandenka)	Africa	12.00	23	0.040	[2]
North American Inuit	Americas	65.00	350	0.180	[4]
Mexican - Maya	Americas	19.00	102	0.180	[4]
Peru - Quechua	Americas	-16.56	208	0.240	[4]
CEPH - Brazil (Surui)	Americas	-11.00	21	0.000	[2]
CEPH - Brazil (Karitiana)	Americas	-10.00	24	0.000	[2]
CEPH - Colombia	Americas	3.00	13	0.150	[2]
CEPH - Mexico (Pima)	Americas	29.00	25	0.100	[2]
CEPH - Mexico (Maya)	Americas	19.00	25	0.080	[2]
CEPH - China (Uygur)	Central/South Asia	44.00	10	0.600	[2]
CEPH - Pakistan (Hazara)	Central/South Asia	33.50	24	0.270	[2]
CEPH - Pakistan (Kalash)	Central/South Asia	35.99	25	0.300	[2]
CEPH - Pakistan (Burusho)	Central/South Asia	36.50	24	0.230	[2]
CEPH - Pakistan (Balochi)	Central/South Asia	30.50	21	0.430	[2]
CEPH - Pakistan (Sindhi)	Central/South Asia	25.49	25	0.160	[2]
CEPH - Pakistan (Makrani)	Central/South Asia	26.00	25	0.260	[2]
CEPH - Pakistan (Brahui)	Central/South Asia	30.50	25	0.460	[2]
CEPH - Pakistan (Pathan)	Central/South Asia	33.49	24	0.250	[2]
Chinese - Han	East Asia	32.27	414	0.130	[4]
Japanese (JBIC-allele)	East Asia	38.00	552	0.188	[3]
IMCJ-JP1- EAST ASIA (Japanese)	East Asia	38.00	130	0.200	[3]
IMCJ-JP2- EAST ASIA (Japanese)	East Asia	38.00	88	0.159	[3]
CEPH - China (Dai)	East Asia	21.00	10	0.100	[2]
CEPH - China (She)	East Asia	27.00	10	0.200	[2]
CEPH - China (Miaozu)	East Asia	28.00	10	0.150	[2]
CEPH - China (Tujia)	East Asia	29.00	10	0.150	[2]
CEPH - China (Mongolia)	East Asia	45.00	10	0.200	[2]
CEPH - China (Naxi)	East Asia	26.00	10	0.150	[2]
CEPH - China (Lahu)	East Asia	22.00	10	0.050	[2]
CEPH - China (Han)	East Asia	32.27	44	0.160	[2]
CEPH - China (Xibo)	East Asia	43.50	9	0.330	[2]
CEPH - China (Yizu)	East Asia	28.00	10	0.250	[2]
CEPH - China (Hezhen)	East Asia	47.50	10	0.150	[2]
CEPH - China (Daur)	East Asia	48.50	10	0.300	[2]
CEPH - China (Tu)	East Asia	36.00	10	0.200	[2]
CEPH - China (Orogen)	East Asia	50.43	10	0.100	[2]
CEPH - Japan	East Asia	38.00	30	0.270	[2]

CEPH - Cambodia	East Asia	12.00	11	0.140	[2]
CEPH - Siberia (Yakut)	East Asia	62.98	25	0.260	[2]
Czech (Prague)	Europe	50.08	250	0.560	[4]
French	Europe	46.00	1716	0.584	[4]
Spaniards (Madrid)	Europe	40.42	790	0.570	[4]
IMCJ-CA1- NORTH AMERICA (Caucasian)	Europe	~	198	0.611	[3]
CEPH - Italy (Bergamo)	Europe	46.00	14	0.360	[2]
CEPH - Orkney Islands (Orcadian)	Europe	59.00	16	0.560	[2]
CEPH - Russia Caucasus (Adygei)	Europe	44.00	17	0.590	[2]
CEPH - France	Europe	46.00	28	0.660	[2]
CEPH - Russian	Europe	61.00	24	0.560	[2]
CEPH - Italy (Tuscan)	Europe	43.00	7	0.280	[2]
CEPH - Italy (Sardinian)	Europe	40.00	28	0.630	[2]
CEPH - France (Basque)	Europe	43.00	20	0.680	[2]
CEPH - Algeria (Mozabite)	Middle East	32.00	26	0.400	[2]
CEPH - Israel (Palestinian)	Middle East	32.00	50	0.460	[2]
CEPH - Israel (Bedouin)	Middle East	31.00	48	0.440	[2]
CEPH - Israel (Druze)	Middle East	32.00	48	0.440	[2]
CEPH - New Guinea (Papuan)	Oceania	-4.00	17	0.090	[2]
CEPH - Melanesian (Bougainville)	Oceania	-6.00	22	0.050	[2]

Supplementary Table 6. Frequencies of the *GNB3* g.4423C>T minor allele in other worldwide populations.

Population	Region	Latitude	N	MAF (T)	Reference
Zimbabwe (Harare)	Africa	-17.86	299	0.810	[5]
Brazil (Xingu)	Americas	-7.73	25	0.260	[5]
Brazil (Yanomami)	Americas	3.13	25	0.320	[5]
Brazil (Vitoria)	Americas	-20.29	1568	0.450	[6]
Brazil (Caucasian)	Americas	-20.29	548	0.362	[6]
Brazil (Mulatto)	Americas	-20.29	795	0.487	[6]
Brazil (African origin)	Americas	-20.29	122	0.627	[6]
Brazil (Other)	Americas	-20.29	87	0.391	[6]
Pakistan (Islamabad)	Central/South Asia	33.71	211	0.517	[7]
Japan (Osaka)	East Asia	35.95	1640	0.524	[5]
Japan (Tochigi Perfecture)	East Asia	36.38	1640	0.524	[5]
Japan (Toyama)	East Asia	36.68	1640	0.524	[5]
China (Han)	East Asia	32.27	503	0.470	[8]
China (Beijing)	East Asia	39.87	625	0.513	[5]
China (Tianjin)	East Asia	39.13	625	0.513	[5]
China (Nanning)	East Asia	22.82	244	0.430	[5]
South Korea (Pusan)	East Asia	35.70	31	0.440	[5]
Taiwan (San Chilo village)	East Asia	23.35	199	0.543	[5]
US (White, Goodman)	Europe	32.58	1302	0.291	[5]
US (White, Jackson)	Europe	32.32	1302	0.291	[5]
Belgium (Charleroi)	Europe	50.42	1512	0.308	[5]
Belgium (Ghent)	Europe	51.05	1512	0.308	[5]
Finland (Joensuu)	Europe	61.57	100	0.240	[5]
Finland (Turku)	Europe	60.45	100	0.240	[5]
Hungary (Porcsalma Village)	Europe	47.88	27	0.320	[5]
Italy (Bassiano)	Europe	41.55	39	0.300	[5]
Italy (Gubbio)	Europe	43.35	29	0.300	[5]
Italy (Mirano)	Europe	45.49	45	0.320	[5]
Poland (Krakow)	Europe	50.08	60	0.220	[5]
Poland (Warsaw)	Europe	52.25	60	0.220	[5]
Soviet Union (Moscow)	Europe	55.75	53	0.220	[5]
East Germany (Cottbus)	Europe	51.67	1855	0.300	[5]
West Germany (Bernried)	Europe	49.32	1855	0.300	[5]
West Germany (Heidelberg)	Europe	49.42	1855	0.300	[5]
United Kingdom (Belfast)	Europe	54.58	163	0.300	[5]
Spain (Sevilla)	Europe	37.39	78	0.397	[9]
Spain (Manresa)	Europe	41.73	30	0.300	[5]
Spain (Torrejon)	Europe	40.65	30	0.300	[5]
Danish (Copenhagen)	Europe	55.68	4193	0.310	[10]
Papua New Guinea (Asaro Valley)	Oceania	-6.58	47	0.564	[5]
US (African-American, Goodman)		32.58	821	0.260	[5]
US (African American, Jackson)		32.32	821	0.260	[5]
Trinadad and Tobago (Plymouth, Bethesada)		11.22	10	0.850	[5]

Supplementary Table 7. Frequencies of the *CAPN10* g.4834T>C minor allele in other worldwide populations.

Population	Region	N	MAF (C)	Reference
YRI	Africa	60	0.017	[1]
Biaka	Africa	~	0.020	[4]
Mbuti	Africa	~	0.020	[4]
AFD_AFR_PANEL	Africa	23	0.022	[11]
Maya	Americas	~	0.060	[4]
Surui	Americas	~	0.010	[4]
CHB	East Asia	45	0.111	[1]
JPT	East Asia	45	0.089	[1]
Han	East Asia	~	0.100	[4]
Japanese	East Asia	~	0.010	[4]
AFD_CHN_PANEL	East Asia	24	0.063	[11]
Yakut	East Asia	~	0.120	[4]
CEU	Europe	60	0.167	[1]
Druze	Europe	~	0.140	[4]
Danes	Europe	~	0.180	[4]
Europe (Mixed)	Europe	~	0.120	[4]
AFD_EUR_PANEL	Europe	24	0.125	[11]

Supplementary Table 8. Frequencies of the *TCF7L2* g.98386G>T minor allele in other worldwide populations.

Population	Region	N	MAF (T)	Reference
Icelandic	Europe	795	0.288	[12]
Danish	Europe	520	0.261	[12]
US	Europe	509	0.259	[12]
CEU	Europe	60	0.217	[1]
CHB	East Asia	45	0.000	[1]
JPT	East Asia	44	0.023	[1]
YRI	Africa	60	0.267	[1]

Supplementary Table 9. Frequencies of the *PTPN22* g.36677C>T minor allele in other worldwide populations.

Population	Region	N	MAF (T)	Reference
YRI	Africa	60	0.000	[1]
CHB	East Asia	45	0.000	[1]
JPT	East Asia	44	0.000	[1]
UK	Europe	374	0.084	[13]
Spainish	Europe	812	0.062	[14]
German	Europe	214	0.030	[15]
French	Europe	100	0.090	[16]
French	Europe	296	0.074	[17]
Canadian	Europe	791	0.086	[18]
CEU	Europe	60	0.142	[1]
US	Europe	~	0.089	[19]
US	Europe	1178	0.086	[20]
US	Europe	1961	0.086	[21]

Supplementary Table 10. Frequencies of the *ALOX5* g.20C>T minor allele in other worldwide populations.

Population	Region	N	MAF (T)	Reference
Caucasian	Europe	60	0.100	[22]
African	Africa	48	0.000	[22]
Hispanic	Americas	46	0.000	[22]
Pacific	Oceania	46	0.000	[22]

Supplementary Table 11. Frequencies of the *ALOX5* g.8322G>A minor allele in other worldwide populations.

Population	Region	N	MAF (A)	Reference
YRI	Africa	60	0.642	[1]
African	Africa	46	0.348	[23]
African	Africa	60	0.109	[23]
Hispanic	Americas	46	0.087	[23]
CHB	East Asia	45	0.089	[1]
JPT	East Asia	44	0.057	[1]
Chinese	East Asia	48	0.042	[23]
Japanese	East Asia	1482	0.042	[23]
Asian	East Asia	84	0.045	[23]
CEU	Europe	60	0.017	[1]
European	Europe	48	0.021	[23]
Caucasian	Europe	198	0.000	[23]
Pacific	Oceania	46	0.000	[23]

Supplementary Table 12. Frequencies of the *ALOX5* g.50778G>A minor allele in other worldwide populations.

Population	Region	N	MAF (A)	Reference
AFR1	Africa	48	0.083	[24]
African	Africa	118	0.144	[24]
HISP1	Americas	44	0.000	[24]
Japanese	East Asia	1480	0.016	[24]
CAUC1	Europe	62	0.000	[24]
Caucasian	Europe	120	0.000	[24]
PAC1	Oceania	46	0.022	[24]

Supplementary Table 13. Frequencies of the *rs1447295* minor allele in other worldwide populations.

Population	Region	N	MAF (A)	Reference
YRI	Africa	60	0.342	[1]
Asian	East Asia	42	0.150	[25]
CHB	East Asia	45	0.111	[1]
JPT	East Asia	44	0.205	[1]
Japan	East Asia	37	0.149	[25]
Asian	East Asia	24	0.104	[25]
Sweden	Europe	779	0.133	[26]
Iceland	Europe	997	0.106	[26]
US (Caucasian)	Europe	247	0.082	[26]
European	Europe	24	0.083	[25]
CEU	Europe	60	0.067	[1]
Caucasian	Europe	41	0.048	[25]

Supplementary Table 14. Frequencies of the *SLC24A5* g.13242G>A minor allele in other worldwide populations.

Population	Region	N	MAF (G)	Reference
YRI	Africa	60	0.975	[1]
Nigeria	Africa	95	0.980	[27]
Puerto Rican	Americas	95	0.410	[27]
Mexican American	Americas	90	0.500	[27]
Mexican	Americas	94	0.620	[27]
Amerindian (Mayan)	Americas	72	0.950	[27]
CHB	East Asia	45	0.989	[1]
JPT	East Asia	44	0.989	[1]
East Asian	East Asia	80	0.970	[27]
AoD Chinese	East Asia	90	0.990	[27]
CEU	Europe	59	0.000	[1]
AoD Caucasian	Europe	92	0.020	[27]
European American	Europe	114	0.000	[27]

Supplementary Table 15. Frequencies of the *CFH* g.37989T>C minor allele in other worldwide populations.

Population	Region	N	MAF (C)	Reference
YRI	Africa	60	0.307	[1]
CHB	East Asia	45	0.068	[1]
JPT	East Asia	44	0.081	[1]
US	Europe	24	0.542	[28]
US	Europe	202	0.356	[29]
German	Europe	275	0.338	[30]
Icelandic	Europe	171	0.354	[31]
Icelandic	Europe	891	0.389	[31]
Utah	Europe	203	0.399	[31]
French	Europe	91	0.302	[32]
UK	Europe	262	0.363	[33]
CEU	Europe	59	0.390	[1]

Supplementary Table 16. Frequencies of the *LOC381175* g.205G>T minor allele in other worldwide populations.

Population	Region	N	MAF (T)	Reference
YRI	African	60	0.308	[1]
CHB	East Asia	45	0.466	[1]
JPT	East Asia	44	0.420	[1]
Asian	East Asia	10	0.400	[34]
CEU	Europe	60	0.217	[1]
Caucasian	Europe	24	0.167	[34]
European	Europe	12	0.000	[34]

Supplementary Table 17. Frequencies of the *RET* g.9349G>A minor allele in other worldwide populations.

Population	Region	N	MAF (T)	Reference
CEPH-Africa	Africa	~	0.001	[35]
CEPH-Asia	East Asia	~	0.450	[35]
CEPH-Europe	Europe	~	0.250	[35]

Supplementary Table 18. Frequencies of the *TAS2R38* g.144G>C minor allele in other worldwide populations.

Population	Region	N	MAF (C)	Reference
YRI	Africa	60	0.392	[1]
Biaka	Africa	138	0.717	[4]
Hausa	Africa	78	0.436	[4]
Ibo	Africa	96	0.438	[4]
Mbuti	Africa	76	0.539	[4]
Yoruba	Africa	156	0.474	[4]
Chagga	Africa	90	0.556	[4]
Ethiopian	Africa	62	0.500	[4]
Masai	Africa	40	0.525	[4]
Cheyenne	Americas	108	0.380	[4]
Pima (Arizona)	Americas	102	0.039	[4]
Pima (Mexico)	Americas	106	0.019	[4]
Maya (Yucatan)	Americas	96	0.115	[4]
Karitiana	Americas	110	0.000	[4]
Surui	Americas	90	0.244	[4]
Ticuna	Americas	130	0.100	[4]
Khanty	Central/South Asia	100	0.620	[4]
CHB	East Asia	45	0.678	[1]
JPT	East Asia	44	0.534	[1]
Ami	East Asia	80	0.225	[4]
Atayal	East Asia	80	0.450	[4]
Han	East Asia	96	0.302	[4]
Han	East Asia	110	0.327	[4]
Japanese	East Asia	92	0.467	[4]
Korean	East Asia	128	0.453	[4]
Cambodians (Khmer)	East Asia	50	0.360	[4]
Hakka	East Asia	82	0.232	[4]
Yakut	East Asia	102	0.549	[4]
CEU	Europe	60	0.425	[1]
Druze	Europe	204	0.426	[4]
Jews (Yemenite)	Europe	82	0.500	[4]
Samaritans	Europe	80	0.375	[4]
Adygei	Europe	108	0.528	[4]
Chuvash	Europe	82	0.683	[4]
Danes	Europe	102	0.588	[4]
Europeans (Mixed)	Europe	176	0.659	[4]
Finns	Europe	70	0.543	[4]
Irish	Europe	222	0.568	[4]
Jews (Ashkenazi)	Europe	162	0.469	[4]
Russians	Europe	96	0.594	[4]
Komi-Zyrian	Europe	94	0.606	[4]
Melanesian (Nasioi)	Oceania	42	0.190	[4]
Micronesians	Oceania	68	0.250	[4]

Supplementary Table 19. Frequencies of the *TAS2R38* g.784C>T minor allele in other worldwide populations.

Population	Region	N	MAF (C)	Reference
YRI	Africa	60	0.667	[1]
Biaka	Africa	138	0.720	[4]
Hausa	Africa	78	0.750	[4]
Ibo	Africa	96	0.745	[4]
Mbuti	Africa	76	0.878	[4]
Yoruba	Africa	156	0.795	[4]
Chagga	Africa	90	0.756	[4]
Ethiopian	Africa	62	0.554	[4]
Masai	Africa	40	0.750	[4]
Cheyenne	Americas	108	0.627	[4]
Pima (Arizona)	Americas	102	0.959	[4]
Pima (Mexico)	Americas	106	0.981	[4]
Maya (Yucatan)	Americas	96	0.883	[4]
Karitiana	Americas	110	1.000	[4]
Surui	Americas	90	0.756	[4]
Ticuna	Americas	130	0.908	[4]
Khanty	Central/South Asia	100	0.380	[4]
CHB	East Asia	45	0.678	[1]
JPT	East Asia	44	0.534	[1]
Ami	East Asia	80	0.775	[4]
Atayal	East Asia	80	0.550	[4]
Han	East Asia	96	0.689	[4]
Han	East Asia	110	0.667	[4]
Japanese	East Asia	92	0.522	[4]
Korean	East Asia	128	0.547	[4]
Cambodians (Khmer)	East Asia	50	0.640	[4]
Hakka	East Asia	82	0.768	[4]
Yakut	East Asia	102	0.451	[4]
CEU	Europe	60	0.500	[1]
Druze	Europe	204	0.594	[4]
Jews (Yemenite)	Europe	82	0.525	[4]
Samaritans	Europe	80	0.613	[4]
Adygei	Europe	108	0.500	[4]
Chuvash	Europe	82	0.342	[4]
Danes	Europe	102	0.451	[4]
Europeans (Mixed)	Europe	176	0.394	[4]
Finns	Europe	70	0.471	[4]
Irish	Europe	222	0.500	[4]
Jews (Ashkenazi)	Europe	162	0.580	[4]
Russians	Europe	96	0.424	[4]
Komi-Zyrian	Europe	94	0.394	[4]
Melanesian (Nasioi)	Oceania	42	0.800	[4]
Micronesians	Oceania	68	0.743	[4]

Supplementary Table 20. Frequencies of the *TAS2R38* g.885A>G minor allele in other worldwide populations.

Population	Region	N	Allele (G)	Reference
YRI	Africa	60	0.398	[1]
Biaka	Africa	138	0.277	[4]
Hausa	Africa	78	0.579	[4]
Ibo	Africa	96	0.552	[4]
Mbuti	Africa	76	0.621	[4]
Yoruba	Africa	156	0.507	[4]
Chagga	Africa	90	0.443	[4]
Ethiopian	Africa	62	0.519	[4]
Masai	Africa	40	0.500	[4]
Cheyenne	Americas	108	0.640	[4]
Pima (Arizona)	Americas	102	0.960	[4]
Pima (Mexico)	Americas	106	0.990	[4]
Maya (Yucatan)	Americas	96	0.883	[4]
Karitiana	Americas	110	1.000	[4]
Surui	Americas	90	0.761	[4]
Ticuna	Americas	130	0.902	[4]
Khanty	Central/South Asia	100	0.380	[4]
CHB	East Asia	45	0.678	[1]
JPT	East Asia	44	0.537	[1]
Ami	East Asia	80	0.769	[4]
Atayal	East Asia	80	0.550	[4]
Han	East Asia	96	0.696	[4]
Han	East Asia	110	0.667	[4]
Japanese	East Asia	92	0.541	[4]
Korean	East Asia	128	0.546	[4]
Cambodians (Khmer)	East Asia	50	0.667	[4]
Hakka	East Asia	82	0.775	[4]
Yakut	East Asia	102	0.468	[4]
CEU	Europe	60	0.500	[1]
Druze	Europe	204	0.595	[4]
Jews (Yemenite)	Europe	82	0.513	[4]
Samaritans	Europe	80	0.569	[4]
Adygei	Europe	108	0.491	[4]
Chuvash	Europe	82	0.341	[4]
Danes	Europe	102	0.451	[4]
Europeans (Mixed)	Europe	176	0.401	[4]
Finns	Europe	70	0.457	[4]
Irish	Europe	222	0.505	[4]
Jews (Ashkenazi)	Europe	162	0.580	[4]
Russians	Europe	96	0.417	[4]
Komi-Zyrian	Europe	94	0.391	[4]
Melanesian (Nasioi)	Oceania	42	0.806	[4]
Micronesians	Oceania	68	0.759	[4]

Reference

1. The International HapMap C: **A haplotype map of the human genome**. *Nature* 2005, **437**(7063):1299-1320.
2. Thompson EE, Kuttab-Boulos H, Witonsky D, Yang L, Roe BA, Di Rienzo A: ***CYP3A* variation and the evolution of salt-sensitivity variants**. *Am J Hum Genet* 2004, **75**(6):1059-1069.
3. **Database of Single Nucleotide Polymorphisms (dbSNP). Bethesda (MD): National Center for Biotechnology Information, National Library of Medicine. dbSNP accession: ss11901, ss16338660, ss16338962, ss20420720, ss22886629, ss3240877, ss48405656, & ss6484, (dbSNP Build ID: 126).** [http://www.ncbi.nlm.nih.gov/SNP/]
4. Osier MV, Cheung K-H, Kidd JR, Pakstis AJ, Miller PL, Kidd KK: **ALFRED: An allele frequency database for anthropology**. *American Journal of Physical Anthropology* 2002, **119**(1):77-83.
5. Young JH, Chang Y-PC, Kim JD-O, Chretien J-P, Klag MJ, Levine MA, Ruff CB, Wang N-Y, Chakravarti A: **Differential Susceptibility to Hypertension Is Due to Selection during the Out-of-Africa Expansion**. *PLoS Genetics* 2005, **1**(6):e82.
6. Danoviz ME, Pereira AC, Mill JG, Krieger JE: **Hypertension, Obesity and *GNB3* gene variants.** *Clin Exp Pharmacol Physiol* 2006, **33**(3):248-252.
7. Mahmood MS, Mian ZS, Afzal AF, P.M.: **G-protein beta-3 subunit gene 825C>T dimorphism is associated with left ventricular hypertrophy but not essential hypertension.** *Med Sci Monit* 2005, **11**(1):CR6-CR9.
8. Li B, Ge D, Wang Y, Zhao W, Zhou X, Gu D, Chen R: **G Protein B3 Subunit Gene Variants and Essential Hypertension in the Northern Chinese Han Population**. *Annals of Human Genetics* 2005, **69**(4):468-473.
9. Martin DN, Andreu EP, Ramirez Lorca R, Garcia-Junco PS, Vallejo Maroto I, Santos RA, Miranda Guisado ML, Grijalvo OM, Ortiz JV, Carneado de la Fuente J: **G-protein beta-3 subunit gene C825 T polymorphism: Influence on plasma sodium and potassium concentrations in essential hypertensive patients**. *Life Sciences* 2005, **77**(23):2879.
10. Andersen G, Overgaard J, Albrechtsen A, GlÃ¼mer C, Borch-Johnsen K, JÃ¸rgensen T, Hansen T, Pedersen O: **Studies of the association of the GNB3 825C>T polymorphism with components of the metabolic syndrome in white Danes**. *Diabetologia* 2006, **49**(1):75.

11. **Database of Single Nucleotide Polymorphisms (dbSNP). Bethesda (MD): National Center for Biotechnology Information, National Library of Medicine. dbSNP accession: ss24654536 & ss5319242, (dbSNP Build ID: 126).** [http://www.ncbi.nlm.nih.gov/SNP/]

12. Grant SFA, Thorleifsson G, Reynisdottir I, Benediktsson R, Manolescu A, Sainz J, Helgason A, Stefansson H, Emilsson V, Helgadottir A *et al*: **Variant of transcription factor 7-like 2 (TCF7L2) gene confers risk of type 2 diabetes**. *Nat Genet* 2006, **38**(3):320.

13. Prescott NJ, Fisher SA, Onnie C, Pattni R, Steer S, Sanderson J, Forbes A, Lewis CM, Mathew CG: **A general autoimmunity gene (PTPN22) is not associated with inflammatory bowel disease in a British population**. *Tissue Antigens* 2005, **66**(4):318-320.

14. Martin MC, Oliver J, Urcelay E, Orozco G, Gomez-Garcia M, Lopez-Nevot MA, Pinero A, Brieva JA, de la Concha EG, Nieto A *et al*: **The functional genetic variation in the PTPN22 gene has a negligible effect on the susceptibility to develop inflammatory bowel disease**. *Tissue Antigens* 2005, **66**(4):314-317.

15. Wagenleiter SEN, Klein W, Griga T, Schmiegel W, Epplen JT, Jagiello P: **A case-control study of tyrosine phosphatase (PTPN22) confirms the lack of association with Crohn's disease**. *International Journal of Immunogenetics* 2005, **32**(5):323-324.

16. Wipff J, Allanore Y, Kahan A, Meyer O, Mouthon L, Guillevin L, Pierlot C, Glickmans E, Bardin T, Boileau C *et al*: **Lack of association between the protein tyrosine phosphatase non receptor 22 (PTPN22) - 620W Allele and systemic sclerosis in the French Caucasian population**. *Ann Rheum Dis* 2006:ard.2005.048181.

17. Vandiedonck C, Capdevielle C, Giraud M, Krumeich S, Jais J-P, Eymard B, ChristineTranchant, Gajdos P, Garchon H-J: **Association of the PTPN22*R620W polymorphism with autoimmune myasthenia gravis**. *Annals of Neurology* 2006, **59**(2):404-407.

18. Van-Oene M, Wintle RF, Liu X, Yazdanpanah M, Gu X, Newman B, Kwan A, Johnson B, Owen J, Greer W *et al*: **Association of the lymphoid tyrosine phosphatase R620W variant with rheumatoid arthritis, but not Crohn's disease, in Canadian populations**. *Arthritis & Rheumatism* 2005, **52**(7):1993-1998.

19. Carlton VEH, Hu X, Chokkalingam AP, Schrodi SJ, Brandon R, Alexander HC, Chang M, Catanese JJ, Leong DU, Ardlie KG *et al*: **PTPN22 Genetic Variation: Evidence for**

Multiple Variants Associated with Rheumatoid Arthritis. *Am J Hum Genet* 2005, **77**(4):567-581.

20. Zheng W, She J-X: **Genetic Association Between a Lymphoid Tyrosine Phosphatase (PTPN22) and Type 1 Diabetes**. *Diabetes* 2005, **54**(3):906-908.

21. Kyogoku C, Langefeld CD, Ortmann WA, Lee A, Selby S, Carlton VE, Chang MR, Baechler, E.C., Batliwalla FM, Novitzke J, Williams AH *et al*: **Genetic association of the R620W polymorphism of protein tyrosine phosphatase PTPN22 with human SLE.** *Àm J Hum Genet* 2004, **75**(3):504-507.

22. **Database of Single Nucleotide Polymorphisms (dbSNP). Bethesda (MD): National Center for Biotechnology Information, National Library of Medicine. dbSNP accession: ss6903648, (dbSNP Build ID: 126).** [http://www.ncbi.nlm.nih.gov/SNP/]

23. **Database of Single Nucleotide Polymorphisms (dbSNP). Bethesda (MD): National Center for Biotechnology Information, National Library of Medicine. dbSNP accession: ss17401594, ss23587270, ss3172699, ss3250631, ss35036713, & ss5586237, (dbSNP Build ID: 126).** [http://www.ncbi.nlm.nih.gov/SNP/]

24. **Database of Single Nucleotide Polymorphisms (dbSNP). Bethesda (MD): National Center for Biotechnology Information, National Library of Medicine. dbSNP accession: ss3172700, ss35036695, ss48416871, ss4926109, & ss5586239, (dbSNP Build ID: 126).** [http://www.ncbi.nlm.nih.gov/SNP/]

25. **Database of Single Nucleotide Polymorphisms (dbSNP). Bethesda (MD): National Center for Biotechnology Information, National Library of Medicine. dbSNP accession: ss2261839 & ss23360032, (dbSNP Build ID: 126).** [http://www.ncbi.nlm.nih.gov/SNP/]

26. Amundadottir LT, Sulem P, Gudmundsson J, Helgason A, Baker A, Agnarsson BA, Sigurdsson A, Benediktsdottir KR, Cazier J-B, Sainz J *et al*: **A common variant associated with prostate cancer in European and African populations**. *Nat Genet* 2006, **38**(6):652.

27. **Database of Single Nucleotide Polymorphisms (dbSNP). Bethesda (MD): National Center for Biotechnology Information, National Library of Medicine. dbSNP accession: ss2233714 & ss43742806, (dbSNP Build ID: 126).** [http://www.ncbi.nlm.nih.gov/SNP/]

28. Haines JL, Hauser MA, Schmidt S, Scott WK, Olson LM, Gallins P, Spencer KL, Kwan SY, Noureddine M, Gilbert JR *et al*: **Complement Factor H Variant Increases the Risk of Age-Related Macular Degeneration**. *Science* 2005, **308**(5720):419-421.

29. Edwards AO, Ritter R, III, Abel KJ, Manning A, Panhuysen C, Farrer LA: **Complement Factor H Polymorphism and Age-Related Macular Degeneration**. *Science* 2005, **308**(5720):421-424.

30. Scholl H, Weber B, then M, Wienker T, Holz F: **Y402H-Polymorphismus im Komplementfaktor H und altersabhängige Makuladegeneration (AMD)**. *Der Ophthalmologe* 2005, **102**(11):1029.

31. Magnusson KP, Duan S, Sigurdsson H, Petursson H, Yang Z, Zhao Y, Bernstein PS, Ge J, Jonasson F, Stefansson E *et al*: **CFH Y402H Confers Similar Risk of Soft Drusen and Both Forms of Advanced AMD**. *PLoS Medicine* 2006, **3**(1).

32. Souied EH, Leveziel N, Richard F, Dragon-Durey MA, Coscas G, Soubrane G, Benlian P, Fremeaux-Bacchi V: **Y402H complement factor H polymorphism associated with exudative age-related macular degeneration in the French population.** *Mol Vis* 2005, **11**:1135-1140.

33. Sepp T, Khan JC, Thurlby DA, Shahid H, Clayton DG, Moore AT, Bird AC, Yates JRW, the Genetic Factors in AMDSG: **Complement Factor H Variant Y402H Is a Major Risk Determinant for Geographic Atrophy and Choroidal Neovascularization in Smokers and Nonsmokers**. *Invest Ophthalmol Vis Sci* 2006, **47**(2):536-540.

34. **Database of Single Nucleotide Polymorphisms (dbSNP). Bethesda (MD): National Center for Biotechnology Information, National Library of Medicine. dbSNP accession: ss14878415, (dbSNP Build ID: 126).** [http://www.ncbi.nlm.nih.gov/SNP/]

35. Emison ES, McCallion AS, Kashuk CS, Bush RT, Grice E, Lin S, Portnoy ME, Cutler DJ, Green ED, Chakravarti A: **A common sex-dependent mutation in a RET enhancer underlies Hirschsprung disease risk**. *Nature* 2005, **434**(7035):857.
