# Supplementary material for: Prevalence of common disease-associated variants in Asian Indians
Source: BMC Genet. 2008 Feb 4;9:13. doi: 10.1186/1471-2156-9-13 (PMC2267478; doi:10.1186/1471-2156-9-13)
Supplement: Additional file 2 — Supplementary Tables 1–3. Genotype frequencies of the 17 SNPs (Table S1), and the ALOX5P (Table S2) and DG8S737 (Table S3) microsatellites. [file 1471-2156-9-13-S2.pdf]

Supplemental Table 1. Genotype frequencies of the 17 single nucleotide polymorphisms.

Gene Name	Nucleotide Change	Homozygote Major Allele	Frequency	Homozygote Minor Allele	Frequency	Heterozygote	Frequency
AGT	g.802T>C	TT	0.155	CC	0.380	TC	0.465
CYP3A5	g.6980G>A	GG	0.563	AA	0.083	GA	0.354
GNB3	g.4423C>T	CC	0.464	TT	0.100	CT	0.436
ALOX5	g.20C>T	CC	0.668	TT	0.054	CT	0.278
ALOX5	g.8322G>A	GG	0.967	AA	0.000	GA	0.033
ALOX5	g.50778G>A	GG	0.991	AA	0.000	GA	0.009
CAPN10	g.4834T>C	TT	0.596	CC	0.057	TC	0.347
TCF7L2	g.98386G>T	GG	0.565	TT	0.063	GT	0.372
PTPN22	g.36677C>T	CC	0.983	TT	0.000	CT	0.017
rs1447295	C>A	CC	0.760	AA	0.019	CA	0.221
CFH	g.37989T>C	TT	0.511	CC	0.071	TC	0.418
LOC387715	g.205G>T	GG	0.432	TT	0.108	GT	0.460
RET	g.9349G>A	GG	0.567	AA	0.066	GA	0.367
TAS2R38	g.144G>C	GG	0.420	CC	0.120	GC	0.460
TAS2R38	g.784C>T	CC	0.406	TT	0.127	CT	0.467
TAS2R38	g.885A>G	AA	0.401	GG	0.137	AG	0.462
SLC24A5	g.13233G>A	GG	0.790	AA	0.017	GA	0.193

Supplemental Table 2. Genotype frequencies of the ALOX5P (5'-GGGCGG-3')$_{3-8}$ microsatellite

Allele	3	4	5	6	7	8
3	0.002					
4	0.002	0.052				
5	0.007	0.261	0.564			
6		0.016	0.089	0.003		
7			0.002			
8			0.002			

Supplemental Table 3. Genotype frequencies of the DG8S737 (AC)$_{13-30}$ microsatellite

Allele	-10	-9	-8	-7	-6	-5	-4	-3	-2	-1	0	+1	+2	+3	+4	+5	+6	+7
-10																		
-9																		
-8																		
-7	0.002																	
-6																		
-5					0.002													
-4		0.003				0.002	0.014											
-3							0.010											
-2		0.009	0.003		0.002	0.007	0.019	0.019	0.024									
-1	0.002	0.009	0.002		0.009	0.005	0.038	0.028	0.057	0.069								
0		0.002			0.003	0.002	0.036	0.012	0.031	0.059	0.019							
+1	0.002				0.002	0.003	0.012	0.016	0.038	0.036	0.023	0.010						
+2							0.007	0.005	0.017	0.028	0.014	0.003	0.010					
+3		0.003			0.009	0.003	0.010	0.007	0.023	0.052	0.019	0.021	0.003	0.016				
+4		0.002		0.002	0.002			0.003	0.009	0.017	0.016	0.012	0.003	0.002	0.005			
+5					0.003				0.002	0.009	0.003	0.002	0.002	0.003				
+6							0.003					0.002						
+7									0.002			0.002						
